# Exploring the Influence of Ionic Liquid Anion Structure on Gas-Ionic Liquid Partition Coefficients of Organic Solutes Using Machine Learning

**DOI:** 10.1021/acs.langmuir.4c02628

**Published:** 2024-10-30

**Authors:** Karl Marti Toots, Sulev Sild, Jaan Leis, William E. Acree, Uko Maran

**Affiliations:** † Department of Chemistry, University of Tartu, 14a Ravila Street, Tartu 50411, Estonia; ‡ Department of Chemistry, University of North Texas, 1155 Union Circle Drive #305070, Denton, Texas 76203-5017, United States

## Abstract

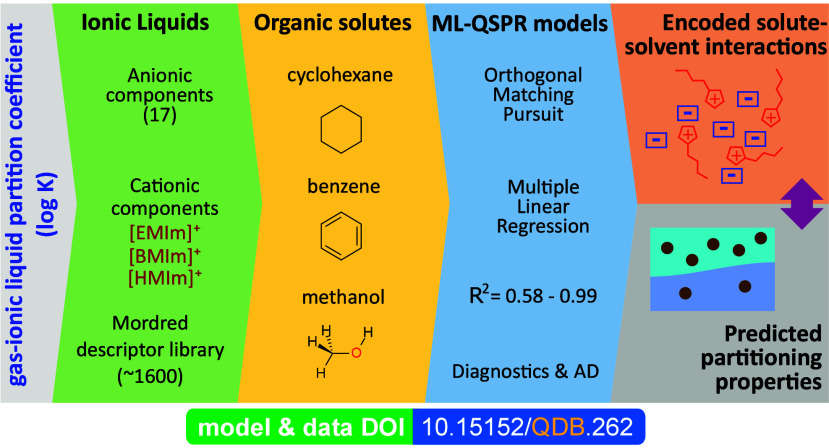

This article presents an in-depth investigation into the influence of anionic structures of ionic liquids (ILs) on gas-ionic liquid partition coefficients (log *K*) of organic solutes in three ILs. While the primary objective was to examine whether there is a relationship between the molecular structure of the IL anion component and log *K*, additionally it was looked at whether the molecular descriptors of the anion in the relationships encode possible molecular interactions during the miscibility and partitioning in the IL. The research involves the compilation of data series of experimental log *K* values, where the cation component is constant. Such representative data series were obtained for three solutes—benzene, cyclohexane, and methanol—in three ILs with a uniform cationic component of methylimidazoliums. Using multiple linear regression models enhanced with machine learning techniques, the relationship between anionic structures and log *K* values was successfully quantified and modeled. Systematically selected molecular descriptors describing the anion structure show that in the case of methanol log *K* is strongly dependent on hydrogen bonds and Coulomb-dipolar interactions with the anion component, while in the case of benzene and cyclohexane the dispersion forces of the anion component are dominant. The outlier analysis and data interpretation highlight the need for extensive experimental data. The results confirm the initial hypothesis and provide valuable information on the role of the structure of the anionic component in determining the partitioning behavior of organic solutes. This knowledge is important for the design and optimization of ILs for specific applications, particularly as solvents in various industrial processes. The research also provides useful information about molecular interactions taking place in the interfaces of IL and organic additives in complex liquid media such as multicomponent electrolyte solutions, for example in energy storage applications.

## Introduction

Ionic liquids (ILs) constitute a distinct class of chemical compounds composed of ions and are recognized as organic salts characterized by their low melting points.^1^ These unique substances possess remarkable properties, including exceptionally low vapor pressure, high polarity, and outstanding thermal stability.^1^ These properties have given rise to extensive research into their utility as green solvents, electrolytes, and a variety of applications in chemical synthesis,^2,3^ catalysis,^2,4−6^ electrochemistry,^7−10^ extraction,^11,12^ energy storage^13−18^ and separation chemistry,^19−21^ where various miscibility and partitioning interactions at IL interfaces play an important role.

Ionic liquids and their interfaces are complex molecular systems, which are often applied together with other organic compounds. The partitioning of organic solvents between the gas and liquid phases becomes an important factor because the presence of impurities or organic additives can change the properties of ILs.^22^ The addition of organic compounds reduces the attraction between counterions of IL, thereby reducing viscosity^23^ and increasing the mobility of IL ions, which is especially important for using ILs as solvents or electrolytes at lower temperatures.^24,25^ The partitioning of additives between gas and IL phases also becomes an essential parameter for evaluating chemical safety considerations. Thus, the gas-IL partition coefficients, *K*, of organic compounds involved become of primary importance in understanding such systems and interfaces. *K* quantifies the distribution of a chemical compound between a gaseous phase and an IL,^26^
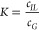
1where *c*_*G*_ and *c*_*IL*_ represent the concentrations of the compound in the gas phase and the IL, respectively. Frequently, this coefficient is represented in its logarithmic form, log *K*. The determination of *K* by experimental methods, such as inverse gas–liquid chromatography (GLC), is labor-intensive, costly, slow, and requires ample amounts of pure compounds. To expedite the virtual screening of compounds for application-specific log *K* values, theoretical and computational machine learning approaches in the frame of Quantitative Structure–Property Relationships (QSPRs), have proven valuable and practical.^27−29^

For molecular interactions affecting the partitioning of an organic compound between an ionic liquid and gas phase, at least three components must be considered: the structure of the organic compound, and the structure of both the cationic and the anionic components of an IL. According to the literature, the partitioning between the gas phase and the IL (log *K*) has been predominantly modeled as a function of the structure of organic compounds only. Examples include the well-known Abraham solvation model^30−41^ and various linear and nonlinear QSPR approaches.^42,43^ Our own recent work in this direction focused on the applying machine learning approaches for the modeling of series organic compounds in three ILs: N-butyl-*N*-methylpyrrolidinium tris(pentafluoroethyl)trifluorophosphate ([BMPyrr]^+^[FAP]^−^), N-butyl-*N*-methylpyrrolidinium tricyanomethanide ([BMPyrr]^+^[C(CN)_3_]^−^) and 1-(2-methoxyethyl)-1-methylpyrrolidinium tris(pentafluoroethyl)trifluorophosphate ([MeoeMPyrr]^+^[FAP]^−^).^44^ Next to this, it is equally important to explain the influence of the structural components of the IL on the partitioning properties. Consequently, characterizing the impact of the charged counterions of the IL and constructing gas-IL partition coefficient models predicted from the molecular structure of ionic components is pivotal for understanding molecular mechanisms in partitioning process and the efficient design of application-tailored ILs. Still, models predicting log *K* in the context of the ionic liquid’s molecular structure remain scarce, with the ion-specific Abraham model^36,40,45−47^ being the only example for a long time. Another example is our recent study that developed machine learning Gaussian process regression (GPR), support vector regression (SVR) and multiple linear regression (MLR) models for a set of ILs where the cationic structure varied.^48^ All systems modeled in this study shared a common anion part (bis(trifluoromethylsulfonyl)-imide), when the partitioning of three hydrocarbons (benzene, hexane and cyclohexane) was investigated. However, how the structure of the anionic component of IL influences the partitioning of the organic compound in the gas-IL system (log *K*) has not been studied before in machine learning models, which is why it is an unexplored area to study.

The present study tests the hypothesis that (a) the gas-IL partition coefficient of organic solutes has a significant dependence on the structure of the anionic component of the IL, and (b) that the relationship between the property and the structure of the anion can be modeled. To explore this the literature was analyzed and data sets were constructed where structure of the organic compounds and the structure of the cationic part of the ILs are constant. Constructed data sets were the subject to analysis with machine learning techniques. Investigating the role of the molecular structure of the anion and its analogues allows the identification of most important structural determinants of anions and interaction mechanisms they are responsible for in the partition process, possibly providing hints and guidelines for the optimization of the anionic component according to the application.

## Materials and Methods

### Data Set

**Figure 1:**
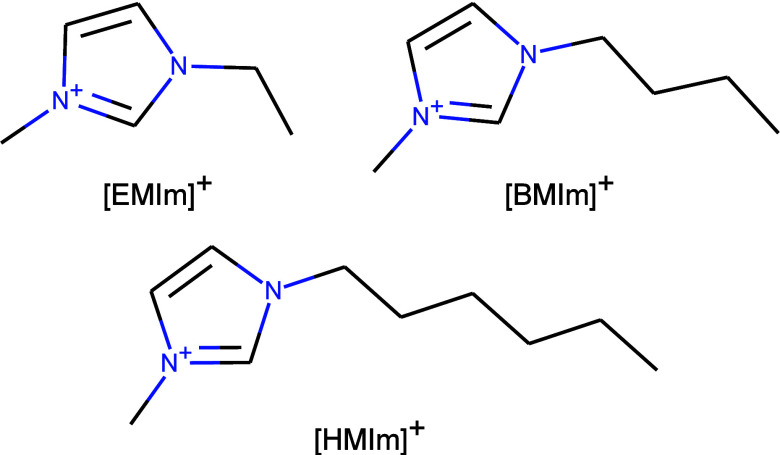
Structures of three cationic components in ionic liquids and their name abbreviations.

**Figure 2:**
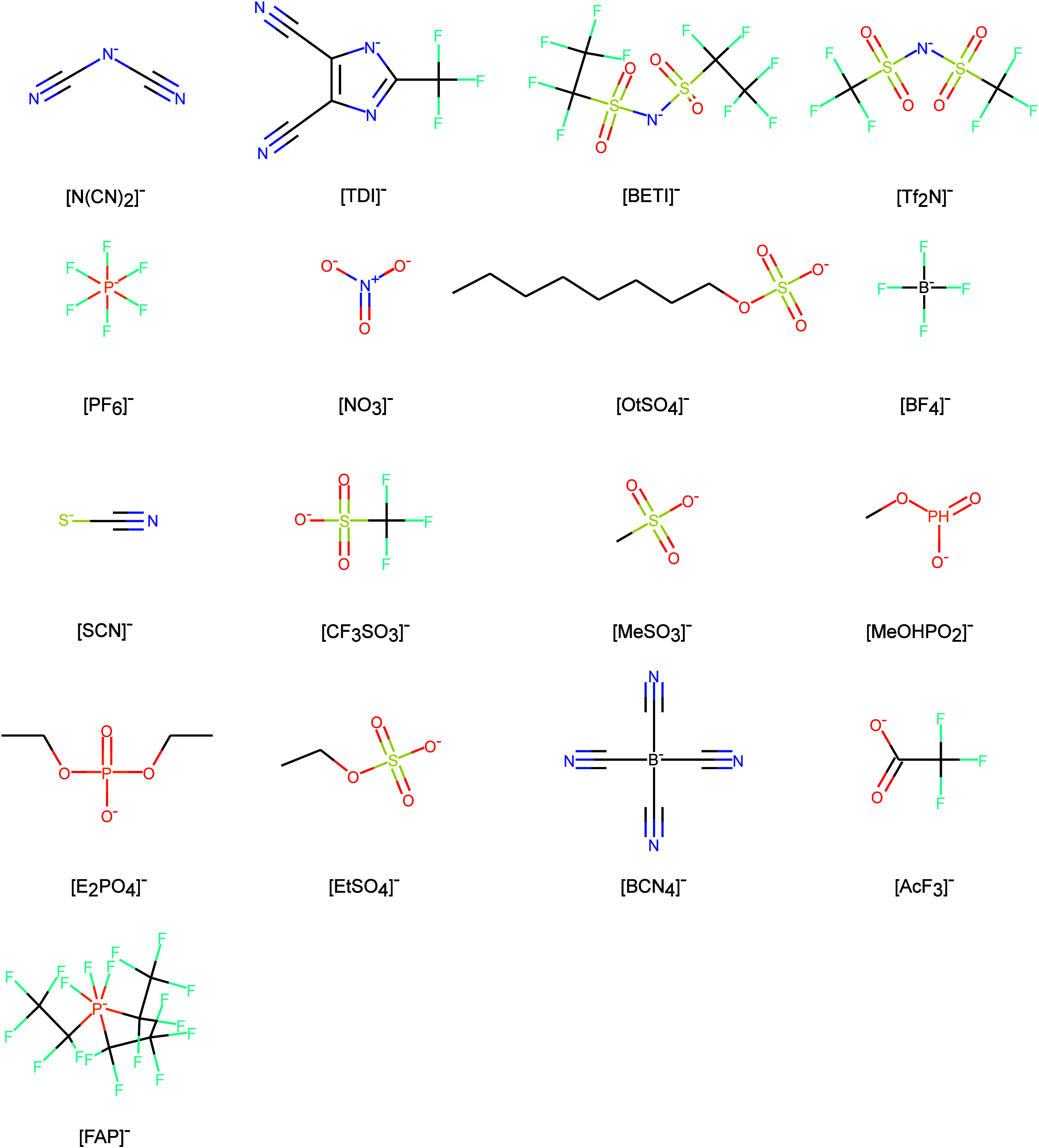
Structures of anionic components in ionic liquids and their name abbreviations.

The review of data sources showed that it is difficult to compile a sizable data series for the modeling, which would include ILs with variable anions but the same cation. The reason lies in the fact that the modification of the anion part of IL is generally less common in the literature, partly because, unlike the anion, the modification of the cation can be more likely targeted at specific ion properties, such as size, shape or charge distribution. Nevertheless, the collected data comprises nine series of experimental gas-IL partition coefficients (log *K*) values at 298 K. Each data series had a common cation and solute, while the anion varied. Collected data series contained one of three cations: ethylmethylimidazolium [EMIm]^+^, butylmethylimidazolium [BMIm]^+^, or hexylmethylimidazolium [HMIm]^+^ (Figure 1) and one of three solutes: benzene, cyclohexane, and methanol (Table 1). The imidazolium ILs chosen for this study have previously gained significant attention in various applications and are preferred for electrosorption processes.^13,49^ These nine data series varied in size (7–13 data points, Table 2) and featured diverse anions with distinct molecular structures, including ionic counterparts, functional groups, symmetricity, heteroatoms, charge distribution, size, and shape (Figure 2). The diversity of the anionic part makes these data series very interesting and challenging to study and model. The log *K* values ranged from 2.232 to 4.366 for methanol, 0.853 to 2.590 for cyclohexane, and 2.297 to 2.977 for benzene, offering a diverse basis for investigating the partitioning behavior of these organic compounds in IL environments.

**Table 1:** Experimental log *K* Values of Solutes Benzene (b), Cyclohexane (c), Methanol (m) in Constant Cation Series with Varying Anions^a^

	Cation									
	**[EMIm]**^**+**^			**[BMIm]**^**+**^			**[HMIm]**^**+**^			
	**Solute**									
Anion	**b**	**c**	**m**	**b**	**c**	**m**	**b**	**c**	**m**	References^b^
**[MeSO**_**3**_**]**^**–**^	2.297	0.853	3.571	2.216*	1.456*	3.339*	2.602*	2.079*	3.033*	(50)
**[(MeO)(H)PO**_**2**_**]**^**–**^	2.430	0.920	2.900	2.760*	1.512*	3.339*	2.537*	1.868*	2.992*	(51)
**[NO**_**3**_**]**^**–**^	2.431	0.959	3.229	2.787	1.454	3.416	2.718	1.840*	3.273	(52−54)
**[SCN]**^**–**^	2.490	0.956	3.208	2.696	1.217	3.280	2.670	1.487	3.101	(55−57)
**[EtSO**_**4**_**]**^**–**^	2.520	1.342	3.254	2.923*	1.684*	3.407*	2.562*	1.941*	2.741*	(58,59)
**[BETI]**^**–**^	3.012*	2.325*	2.470*	2.556	1.614	2.263	2.934*	2.032*	2.129*	(60)
**[AcF**_**3**_**]**^**–**^	2.614	1.442	3.613	2.767*	1.556*	3.078*	2.562	1.728*	3.795	(61,62)
**[N(CN)**_**2**_**]**^**–**^	2.593	1.214	3.207	2.773	1.551	3.297	2.724*	2.160*	3.154*	(63,64)
**[BF4]**^**–**^	2.738	1.361	3.364	2.615	1.475	2.749	2.965	1.824	2.937	(65−68)
**[CF**_**3**_**SO**_**3**_**]**^**–**^	2.677	1.434	3.001	2.692	1.570	2.978	2.734	1.828	3.662*	(69−71)
**[Tf**_**2**_**N]**^**–**^	2.812	1.676	2.655	2.907	1.845	2.589	2.886	2.002	2.478	(59,72)
**[PF**_**6**_**]**^**–**^	2.915*	0.636*	3.325*	2.820	1.542	2.583	2.890	1.795	2.500	(73,74)
**[E**_**2**_**PO**_**4**_**]**^**–**^	2.855	1.977	4.366	2.867*	1.859*	3.407*	2.705*	1.978*	2.564*	(75)
**[OtSO**_**4**_**]**^**–**^	3.071*	2.802*	3.486*	2.897	2.590	3.448	2.973*	1.835*	2.403*	(76)
**[B(CN)**_**4**_**]**^**–**^	2.899	1.719	2.791	1.995*	1.771*	3.407*	2.977	1.953	2.728	(77,78)
**[TDI]**^**–**^	2.492*	2.085*	2.947*	2.965	1.991	3.056	2.614*	1.950*	2.372*	(79)
**[FAP]**^**–**^	3.208*	1.682	2.232	2.563	0.939*	1.390*	2.984	1.942	2.333	(60,80)

a Values calculated using the derived models are indicated with an asterisk.

b Reference to the original literature source.

**Table 2:** Sizes of Data Series

	Benzene	Cyclohexane	Methanol
**[EMIm]**^**+**^	12	13	13
**[BMIm]**^**+**^	10	10	10
**[HMIm]**^**+**^	9	7	8

### Characterization of Anionic Structure Components

The preparation of each data series for modeling adhered to a standardized workflow for calculation of anionic structure components. Initially, SMILES representations^81^ were generated for the anion component of the IL from their chemical names. Subsequently, molecular descriptors were computed using the Mordred^82^ library (version 1.1.1), which relies on the rdkit^83^ library (version 2018.09.3). This computation resulted in a vector of 1613 2D molecular descriptors for each anion, forming an anion-descriptor matrix.

The following steps were undertaken to refine the initial set of descriptors for modeling. Redundant descriptors with constant or too many identical (more than four) values were eliminated. Highly correlated descriptors were filtered by keeping one descriptor from each pair of collinear descriptors. Additionally, descriptors with low variability were excluded, as determined using Kmeans^84^ clustering (K = 2) and measuring standard deviation below 5% within the two groups (resulting in nearly binary descriptors). Finally, ATSC autocorrelation descriptors were also omitted, due to their sensitivity to minor structural changes based on exploratory modeling.

The remaining descriptors were standardized to a mean of zero and a standard deviation of one. Consequently, the data preparation process yielded three distinct anion-descriptor matrices, corresponding to benzene, methanol, and cyclohexane, respectively, containing 507, 551, and 494 descriptors. The descriptors were analyzed and categorized based on the molecular interactions occurring at interfaces within solute–solvent systems, consistent with the methodologies used in our previous studies.^44,48^

### Representation of the Relationships and Selection of Descriptors

The choice of modeling method was guided by the composition and size of the data series. Therefore, it was rational to apply Multiple Linear Regression (MLR) as the fundamental analytical tool for establishing a predictive relationship between molecular descriptors and a target property, denoted as *ŷ*. The MLR model can be expressed as follows:^85^
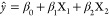
2In the MLR equation, β_0_, β_1_, and β_2_ represent the regression coefficients, while X_1_ and X_2_ denote the two selected molecular descriptors. The aim of MLR is to construct an optimal linear relationship between these descriptors and the modeled property. Other more sophisticated methods were omitted, in comparison with two of our previous studies.^44,48^

The computation of regression coefficients employed the Ordinary Least Squares (OLS) method, which minimizes the squared error. The OLS estimation is calculated using the following formula:
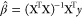
3Here, β̂ signifies the coefficients vector, X corresponds to the matrix of molecular descriptor values, and *y* signifies the vector of experimental property values.^85^

For the feature selection process, the Orthogonal Matching Pursuit (OMP)^86^ algorithm was employed. OMP operates as a bottom-up feature selection approach that iteratively selects molecular descriptors based on their correlation with the residuals from the linear model estimations. It excels when selecting descriptors exhibiting low mutual correlation, thereby enabling the capture of diverse chemical information.^86^

The implementation of OMP was carried out using the OrthogonalMatchingPursuit class from the Scikit-learn library (version 0.24.2).^87^ To adapt the model to the constraints imposed by the limited data set size, the search space of the OMP algorithm was expanded strategically. This involved an iterative removal of the descriptor with the highest correlation to log *K*, followed by a rerun of OMP, until the highest correlation reached a defined threshold of R < 0.4. This iterative process facilitated the generation of a broader selection of linear models.

Among the models with the same number of descriptors identified by the OMP algorithm, the optimal model was chosen based on the highest coefficient of determination. In our case, owing to the small data set size, this process culminated in the selection of two molecular descriptors in the final model, ensuring the establishment of a meaningful and interpretable relationship between the descriptors and the target property.

MLR and step forward molecular descriptor selection algorithms have proven successful in our previous research efforts in this domain and have yielded gas–liquid partition coefficient models applicable to traditional organic solvents^88−91^ and specific solvents like methanol and ethanol.^92^ Moreover, these types of models have found success in various fields, encompassing physicochemical properties,^93,94^ toxicology,^95−97^ biomedicine,^98−100^ and materials science.^101,102^

### Diagnostics and Applicability Domain of Models

The evaluation of model performance involved the utilization of leave-one-out (loo) cross-validation to mitigate the risk of overfitting. In the training process, the model’s predictive capability was assessed through the calculation of the coefficient of determination, *r*^2^ (eq 4). External evaluation, apart from descriptor selection and model generation, was conducted by means of the concordance correlation coefficient, CCC (eq 5).^103^ Additionally, the mean squared error (eq 6), referred to as RMSE, was used in conjunction with the statistical parameters of the models.
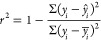
4
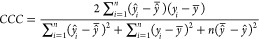
5
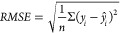
6

The linear models underwent further diagnostic procedures to identify outliers, high leverage data points, and influential data points. Data points exceeding the critical leverage value *h*_3_^*^ (eq 8) were deemed as having high leverage, with the critical value being derived from the model descriptor count (*k*) and the data set size (*n*). The design matrix X, comprising the matrix of model molecular descriptor values in columns along with an additional constant column, was employed to calculate the leverage *h*_*ii*_ of each data point using the hat matrix *H*:
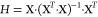
7
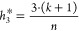
8

For outlier diagnostics, the standardized residuals *r*_*i*_ of the model were examined for each data point *i*:
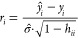
9
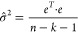
10
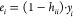
11

A data point with |*r*_*i*_| > 2 warranted inspection, and a data point with |*r*_*i*_| > 3 was likely to be an outlier, necessitating closer analysis. The assessment of an observation’s influence on the model was performed through the computation of Cook’s distance (*D_i_*), which measures the impact of removing a specific data point:
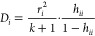
12

It is a common practice to give careful consideration for including observations in the model with Cook’s distance exceeding one. The accuracy of model predictions may be distorted by observations with high leverage and/or high residuals, and Cook’s distance offers a means to identify influential data points that could highlight areas in the molecular space where additional experimental data may be needed.

### Availability of Regression Models

The MLR models and related data can be made available in various data formats.^104^ To follow the best practices of QSAR model reporting^105^ the models with data^106^ are stored at the QsarDB repository^107^ in QSAR Data Bank format.^108^ A digital object identifier (10.15152/QDB.262) has been assigned for the models and data.

## Results and Discussion

During the exploratory modeling, it was found that one parameter models are not sufficient to describe the property, and their statistical metrics are low. To avoid chance correlations and overfitting that may be due to the limited number of data points in each data set, it was decided to derive only models containing two molecular descriptors. This number of descriptors in the model was found to be able to adequately cover the diversity and complexity of structural variability in the data series, for describing log *K* sufficiently, and leaving room for molecular descriptor analysis.

### Benzene in [EMIm]^+^, [BMIm]^+^, [HMIm]^+^ Ionic Liquids

**Figure 3:**
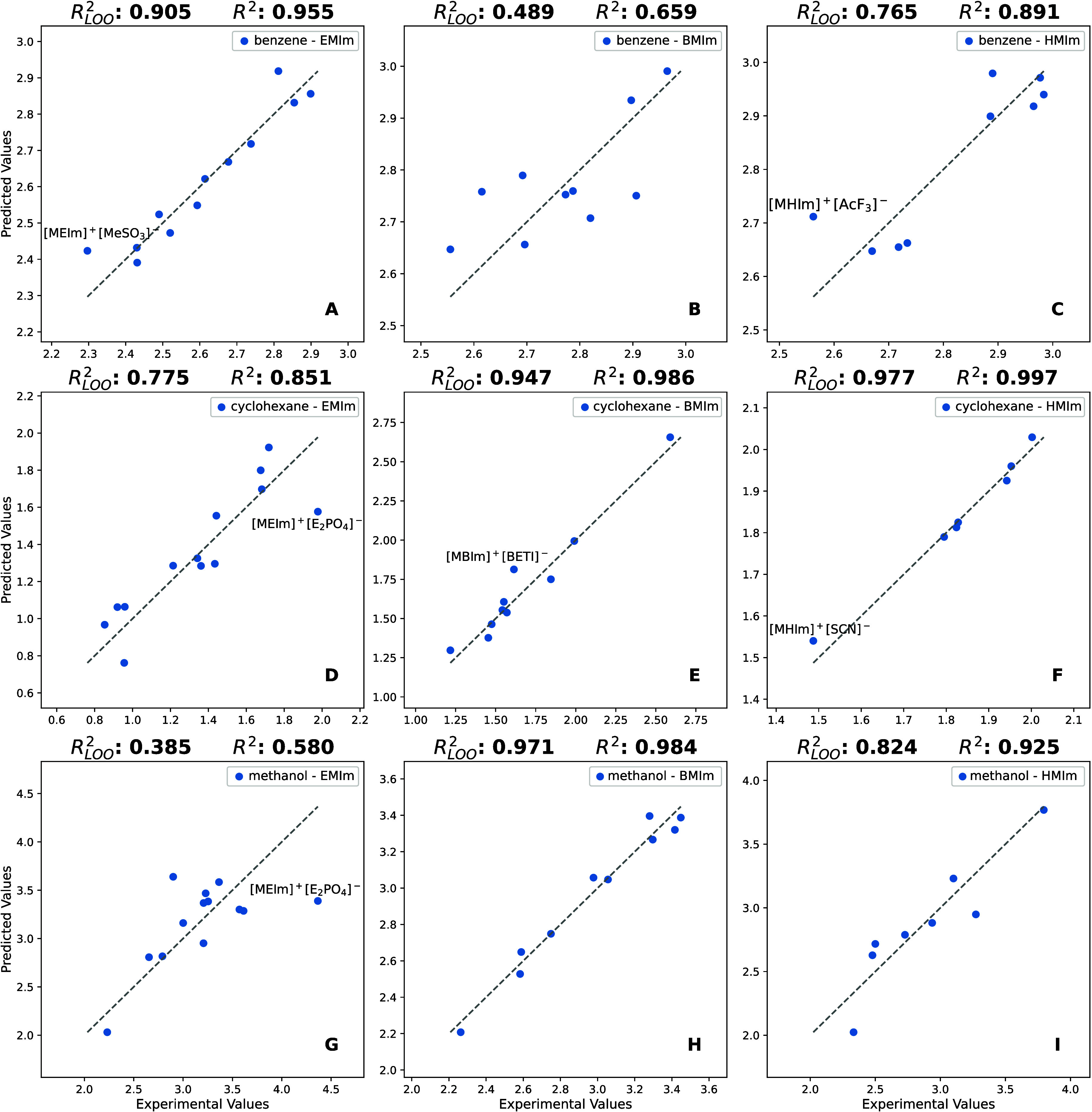
Experimental vs leave-one-out predicted log *K* for each model (10.15152/QDB.262).

The derived model for benzene in [EMIm]^+^ despite a narrow range in experimental values gave a consistent model (eq 13, Figure 3A) according to the fit (*r*^2^ = 0.96) and internal validation (*r*^2^_loo_ = 0.91).
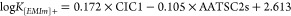
13

The descriptor with the highest standardized regression coefficient in the model is CIC1^109^ (1-ordered complementary information content). The descriptor is based on Shannon entropy^110^ and calculated from atom type counts; it mainly characterizes the anion size and therefore relates to solute–solvent interactions corresponding to the dispersion forces (Table 3). Additionally, more symmetrical anions tend to contain more repeated structural components, resulting in lower Shannon entropy and, consequently, higher descriptor value (Figure S1). The second descriptor in the model, AATSC2s (averaged and centered Moreau-Broto autocorrelation of lag 2 weighted by intrinsic state) is calculated from the average intrinsic electrotopological state and had negative regression coefficient (eq 13). According to the analysis of the structures in the data set, its value increases with the addition of electronegative atoms such as O and N, especially when the functional groups including electronegative atoms placed two bonds apart. Anions with the highest number of oxygen-containing functional groups have the highest AATSC2s values while anions with more fluoride groups have the lowest values (Figure S2). According to solute–solvent interactions both AATSC2s and CIC1 are related to Coulomb and dipolar interactions, AATSC2s is slightly related to hydrogen bonding and CIC1 to dispersion forces (Table 3). It should be mentioned that Basak et al.^109^ in their original publication discussed that CIC1 is related to the hydrophobic interactions (log P) which is consistent with our classification because hydrophobic interactions are determined by size related and electronic effects.

**Table 3:** Descriptor Structural Contribution According to the Solute–Solvent Interactions

		Descriptors		
Interaction types	Main structural contribution	Benzene	Cyclohexane	Methanol
**Dispersion forces** (molecule size, polarizability, molecule shape)	Atom count, volume, chain length	CIC1^a^, IC2^a^, SpMAD_Dzi, MATS3dv^a^	SpMAD_Dzm, SpMAD_D, ATS3m^a^, MATS2m, GATS3dv^a^, AETA_eta_R^a^	VE1_A, AATS3s^a^ ATS0s^a^
	Branching	MATS3dv^a^	GATS3dv^a^, ATS3m^a^	AATS3s^a^
	Lipophilicity^b^	CIC1^a^		
**Coulomb and dipolar interactions** (Charge/electron cloud distribution)	Lipophilicity^b^	CIC1^a^		
	Electronegativity	MATS2se	AETA_eta_R^a^	AETA_beta^a^
	Bond order	MATS3dv^a^	AETA_eta_R^a^	AETA_beta^a^
	Heteroatoms/hydrogen bonding atoms^b^	AATSC2s, CIC1^a^, IC2^a^	ATS3m^a^	AETA_beta^a^, AATS3s^a^, nHetero, nHBAcc, ATS0s^a^
**Hydrogen bonding** (Presence of HB capable heteroatoms)	Heteroatoms/hydrogen bonding atoms^b^	AATSC2s, CIC1^a^, IC2^a^	ATS3m^a^	AETA_beta^a^, AATS3s^a^, nHetero, nHBAcc, ATS0s^a^

a Indicates that the molecular descriptor encodes multiple interaction types.

b Some main structural contributions belong to two interaction types.

The derived model (eq 14) for benzene in [BMIm]^+^ fit (*r*^2^ = 0.67) and internal validation (*r*^2^_loo_ = 0.49) for ten data points (Figure 3B) can be considered reasonably consistent. The CCC_loo_ of 0.698 further validates the consistency of the model (Table 4). The model is strongly influenced by the distribution of data points that may be partially attributed to the quality of the experimental data.
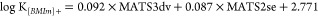
14

**Table 4:** Model Statistics

			Training			loo score		
Equation	Solute	Cation	*CCC*	*RMSE*	*r*^2^	*CCC*	*RMSE*	*r*^2^
13	benzene	[EMIm]^+^	0.978	0.038	0.956	0.951	0.056	0.905
14		[BMIm]^+^	0.800	0.072	0.667	0.698	0.090	0.489
15		[HMIm]^+^	0.946	0.046	0.897	0.877	0.070	0.765
16	cyclohexane	[EMIm]^+^	0.921	0.131	0.854	0.883	0.162	0.775
17		[BMIm]^+^	0.993	0.041	0.987	0.974	0.084	0.947
18		[HMIm]^+^	0.999	0.008	0.997	0.988	0.024	0.977
19	methanol	[EMIm]^+^	0.740	0.320	0.587	0.638	0.391	0.385
20		[BMIm]^+^	0.992	0.048	0.984	0.985	0.066	0.971
21		[HMIm]^+^	0.964	0.121	0.930	0.914	0.192	0.824

The descriptors in the model were MATS3dv (moran coefficient of lag 3 weighted by valence electrons) and MATS2se (moran coefficient of lag 2 weighted by Sanderson electronegativity), both featuring positive regression coefficients (eq 14). The MATS3dv descriptor is grounded in valence electron count products on atom pairs positioned three bonds apart.^111^ Notably, the MATS3dv descriptor value is zero for compact ions without chains (Figure S3). Positive values, on the other hand, may be found on longer chains and similar valence electron count heteroatom pairs (Figure S3). Consequently, this descriptor is primarily associated with Coulomb and dipolar interactions, with a moderate relevance to hydrogen bonding (Table 3). The MATS2se descriptor is calculated based on the products of centered Sanderson electronegativity between atom pairs separated by two bonds within the anion.^111^ This descriptor has a noteworthy trend: its value decreases as the number and ratio of electronegative atoms increases in the ion (Figure S4). The descriptor predominantly derives its value from specific functional groups present in the anions. For instance, the ions with multiple C–SO_3_^–^ or CF_3_ fragments results in more negative values, while the ions with a single SO_4_^–^ and N–N ≡ N fragments yield notably higher positive values. Consequently, similarly to the previous descriptor, the MATS2se descriptor captures the influence of Coulomb and dipolar interactions, along with a consideration of hydrogen bonding effects (Table 3).

The model for benzene within [HMIm]^+^ is also influenced by the availability and quality of the data points (eq 15, Table 4, Figure 3C). Still the fit (*r*^2^ = 0.89), internal validation (*r*^2^_loo_ = 0.77), and CCC of the model are consistent (CCC_loo_ = 0.877).
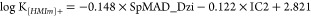
15In the model the two descriptors employed were SpMAD_Dzi (spectral mean absolute deviation from Barysz matrix weighted by ionization potential) and IC2 (2-ordered neighborhood information content). The SpMAD_Dzi descriptor calculation scheme uses the ionization potential of the anion’s constituents,^111^ and its value depends indirectly on the size of the anion (Figure S5). Consequently, this descriptor characterizes dispersion force interactions, while also encompassing considerations of Coulomb and dipolar interactions (Table 3). A negative regression coefficient within the model (eq 15) implies that larger anions, characterized by a greater strength of dispersion force interactions, yield lower log *K* values. On the other hand, the IC2 descriptor is grounded in Shannon entropy,^109^ with higher entropy levels within the anion resulting in elevated descriptor values. This heightened entropy typically arises from an increased count and diversity of functional groups in the anion (Figure S6). As such, in terms of solute–solvent interactions the IC2 bears similarity with the previous descriptors and is primarily associated with Coulomb and dipolar interactions, although it also maintains a modest connection to dispersion force interactions (Table 3).

### Cyclohexane in [EMIm]^+^, [BMIm]^+^, [HMIm]^+^ Ionic Liquids

The cyclohexane model for a series of ILs where the [EMIm]^+^ cation is constant (eq 16, Table 4, Figure 3D) had a reliable fit (*r*^2^ = 0.85) and internal validation (*r*^2^_loo_ = 0.78) supported by a high CCC_loo_ of 0.883.
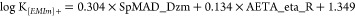
16The model features descriptors SpMAD_Dzm (spectral mean absolute deviation from Barysz matrix weighted by mass) and AETA_eta_R (averaged ETA composite index for reference graph). The SpMAD_Dzm descriptor is founded on the atomic mass of anions^111^ and exhibits a strong correlation with anion size (Figure S7), rendering it particularly pertinent to dispersion force interactions (Table 3). Similarly, the AETA_eta_R descriptor is derived from valence electrons in a structure, where heavy atoms are replaced with single bonded carbons, accounting for size and branching.^112^ This descriptor is therefore related to dispersion interactions (Table 3). The highest log *K* values are estimated with larger anions that have the most branching in the chain, except for the [FAP]^−^ (Figure S8).

The model derived for the cyclohexane data series with constant cation [BMIm]^+^ (eq 17, Table 4, Figure 3E) produced a high fit (*r*^2^ = 0.99) and internal validation (*r*^2^_loo_ = 0.95).
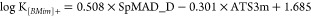
17

The descriptors ATS3m (Moreau-Broto autocorrelation of lag 3 weighted by mass) and SpMAD_D (spectral mean absolute deviation from distance matrix) were chosen for the model. The SpMAD_D descriptor showed notable correlations with various other SpMAD descriptors (Figure S9), as well as anion size and branching characteristics (See Figure S10). This confirms its function in characterizing the contribution to dispersion force interactions within the model (Table 3). Notably, a positive regression coefficient was observed, indicating that higher values of log *K* were associated with enhanced dispersion force interactions (eq 17). On the other hand, the ATS3m descriptor was calculated through the product of molecular masses of atoms situated two bonds apart within the anion.^111^ This descriptor, while displaying a significant correlation with anion size and the number of heteroatoms (Figure S11), was characterized by a negative regression coefficient (eq 17). The ATS3m descriptor has an association with dispersion forces, and a modest association with Coulomb and dipolar interactions and slight connection to hydrogen bonding. Due to a negative regression coefficient, increasing the density of heteroatoms in the anion will lower the predicted log *K* value.

The optimal model for cyclohexane in [HMIm]^+^ had strong internal validation (*r*^2^_loo_ = 0.98) and fit (*r*^2^ = 0.997). The model had the highest metrics (Figure 3F) out of all the derived models.
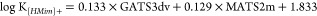
18The model incorporated the descriptors MATS2m (Moran coefficient of lag 2 weighted by mass) and GATS3dv (Geary coefficient of lag 3 weighted by valence electrons). The MATS2m descriptor is based on the molecular masses of the constituent atoms within the anion.^111^ In its calculation, molecular masses are standardized to a zero mean, and it computes the sum of products of standardized molecular masses between atom pairs separated by two bonds within the anion. Consequently, functional groups where atoms at a two-bond distance exhibit significant variation in molecular mass, such as P–F, P–C, S–F, S–C, and S–N, yield negative descriptor values (Figure S12). Anions with predominantly identical or closely matched molecular mass atom pair functional groups like C–C, C–N, C–O, and N–O, exhibit the highest descriptor values. As such, the MATS2m descriptor is primarily associated with Coulomb and dipolar interactions and shows a moderate connection to hydrogen bonding (Table 3). Owing to the positive regression coefficient of the MATS2m descriptor (eq 18), it is projected that smaller anions with a predominant single-atom ionic component will exhibit lower log *K* values, whereas anions containing several similar atom pair-contributing functional groups such as −CSO_3_^–^, BF_4_^–^, and −CSO_2_- are expected to yield higher log *K* values. The GATS3dv descriptor shares similarities and considerable correlation with the MATS3dv descriptor in eq 14 (Figure S9), as it is founded on products of valence electron counts for atom pairs separated by three bonds.^111^ Compact anions without chains remain unimpacted by this parameter, while larger anions with enhanced polarity and a greater number of heteroatoms result in higher descriptor values (Figure S13). The GATS3dv descriptor is primarily associated with Coulomb and dipolar interactions, with a moderate influence from hydrogen bonding (Table 3).

### Methanol in [EMIm]^+^, [BMIm]^+^, [HMIm]^+^ Ionic Liquids

**Figure 4:**
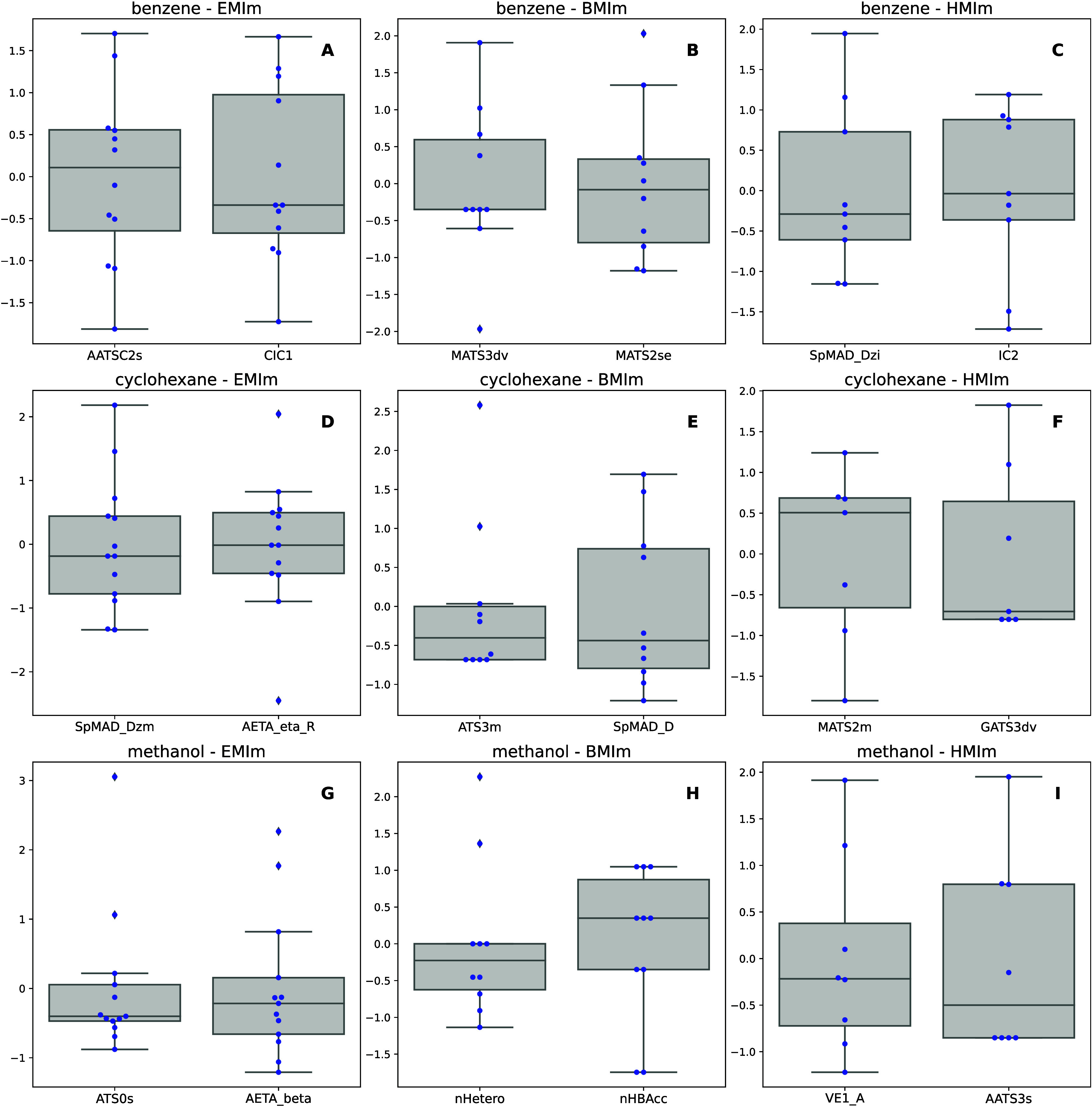
Distribution of molecular descriptor values in each model (box-swarm plots).

The optimal model for methanol within [EMIm]^+^ was impacted by the distribution and quality of the experimental data points. Clustered descriptor data (Figure 4G) and a few outlying experimental data points (Figure 3G) affect the predictive capacity of the model. Despite this, the model had significant fit (*r*^2^ = 0.58), internal validation (*r*^2^_loo_ = 0.39) and a reliable CCC_loo_ of 0.638.
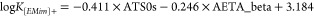
19

In the model, ATS0s (Moreau-Broto autocorrelation of lag 0 weighted by intrinsic state^111^) emerged as the most prominent descriptor, as indicated by its substantial standardized regression coefficient (eq 19). This descriptor is calculated from intrinsic electrotopological states^113^ and it increases in response to the presence of oxygen and nitrogen atoms, as well as atoms with a greater number of attached hydrogens and higher bond order within the anion. Notably, larger anions bearing corresponding functional groups exhibit elevated descriptor values (Figure S14). The model’s negative standardized regression coefficient suggests that it predicts lower log *K* values when larger anions feature functional groups with a propensity for hydrogen bonding and stronger dipolar interactions. The ATS0s descriptor also increases due to greater number of atoms in the ion, linking the descriptor value to strength of dispersion interactions. Furthermore, the AETA_beta descriptor (averaged valence electron mobile count), derived from valence electrons and accounting for bond order and the atomic periodic number,^112^ is closely linked to hydrogen bonding, and moderately related to Coulombic and dipolar interactions. The model predicts the highest log *K* values with larger anions characterized by the presence of the C ≡ N bond, followed by those featuring oxygen-containing functional groups, and finally, fluorine-containing functional groups (Figure S15).

For methanol, the model with the highest fit (*r*^2^ = 0.98) and internal validation (*r*^2^_loo_ = 0.97) was calculated for the constant cation [BMIm]^+^ data series (eq 20, Table 4, Figure 3H).
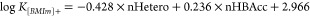
20

In the model, the descriptor with the highest albeit negative regression coefficient value is nHetero, which quantifies the count of heteroatoms (atoms other than C or H) in the anion. Notably, higher values of nHetero were associated with larger anion structures featuring a greater abundance of functional groups. In this data set, such anions were primarily characterized by abundance of fluorine, −SO_2_^–^-, and −SO_3_^–^ groups, all of which possess a propensity for forming multiple hydrogen bond type interactions (Figure S16). The negative standardized regression coefficient of nHetero implies that the model predicts higher log *K* values when the anion is smaller, possesses fewer sites for hydrogen bonding, and exhibits minimal charge disparity due to a lower number of heteroatoms. In these instances, the anions tend to manifest reduced dispersion interactions, fewer hydrogen bonding opportunities, and diminished dipolar interactions. Conversely, the nHBAcc descriptor, which is integrated into the model with a positive standardized regression coefficient, gauges the count of hydrogen bond acceptors.^82^ In this data set, the acceptors present are noncharged O and N atoms within the anion (Figure S17). Notably, the nHBAcc descriptor increases for anions characterized by corresponding functional groups, primarily reflecting their propensity for hydrogen bonding interactions, while also bearing some correlation with Coulomb and dipolar interactions.

Lastly, the model for methanol within [HMIm]^+^ (eq 21, Table 4, Figure 3I) also showed significant fit (*r*^2^ = 0.93) and internal validation (*r*^2^_loo_ = 0.82).
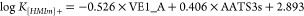
21

In the model, the descriptor with the highest and negative standardized regression coefficient is VE1_A (eq 21), which is directly proportional to a molecule’s size^111^ and is closely associated with dispersion forces (Table 3). This negative relationship with VE1_A (coefficient sum of the last eigenvector of the adjacency matrix) leads to the prediction of higher log *K* values when anions have smaller size (Figure S18). Conversely, the AATS3s descriptor (averaged Moreau-Broto autocorrelation of lag 3 weighted by intrinsic state) exhibits a positive standardized regression coefficient. This descriptor proves particularly relevant for anions of medium to large sizes due to its computation, which involves atom pairs separated by three bonds within the anion structure. The calculation of this descriptor is grounded in the intrinsic electrotopological states of constituent atoms,^111^ akin to ATS0s. Accordingly, higher values of AATS3s descriptor are observed when the anion is predominantly composed of oxygen-containing functional groups, emphasizing its strong correlation with Coulomb and dipolar interactions (Figure S19).

### Physical Interpretation of the Models

Each model contained a unique combination of descriptors, reflecting the specific solute–solvent interaction nuances at interfaces and in the bulk. Although the gas-IL partition coefficient is a bulk property, it is indirectly related to interactions between multiple phases at the interface. It is influenced by how easily the solute molecules transfer across the gas and IL phases, which depends on the physicochemical properties of interacting components. In addition, the miscibility of solute in the IL phase is another factor influencing the partition coefficient. For example, if the solute is not fully miscible with the IL phase, it may form additional interfaces (e.g., dispersion of tiny droplets). The influence of the variation in the anionic structure, coded by molecular descriptors, on the solvent properties of ILs can be understood in the context of general solute–solvent intermolecular interactions, allowing comparison between the final models with different solutes and cations.

The grouping of molecular descriptors (Table 3) according to their relation to solute–solvent interactions allows generalizing which structural properties of the anion in the IL are relevant in the final models. This grouping considers the following general solute–solvent interaction mechanisms: dispersion forces related to molecule size, shape and polarizability; Coulomb and dipolar interactions characterize the charge distribution of anions; and hydrogen bonding interaction is associated with functional groups capable of hydrogen bonding. The presence of all the interaction types across all final models suggests a general mechanism of interaction between the solutes and ILs. However, the degree of fit and internal validation varied across models, indicating differences in the predictive accuracy depending on the cation-solute combination. For benzene and cyclohexane, the molecular descriptors in models across different cations revealed a balanced consideration of various interaction types, with a slight emphasis on dispersion force interactions, i.e. the bulk properties of the anionic structures. All cyclohexane models showed the highest influence from the anion size and branching characteristics, indicating the importance of spatial factors in interactions. In the methanol models, the descriptor selection algorithm consistently favored descriptors related to hydrogen bonding and Coulomb-dipolar interactions, while descriptors related to the dispersion force had negative regression coefficients and had a significant contribution in the [HMIm]^+^ model. This shows that the solubility of methanol in alkylimidazolic ILs is favored if the anion has stronger hydrogen bonding and Coulomb-dipolar interaction capability and is less dependent on dispersion force related molecular features in the anion.

Conversely, the dispersion force has more emphasis in the models for benzene and even more so for cyclohexane. As the cation chain lengthened, there was a noticeable trend toward incorporating descriptors characterizing a broader range of molecular interactions. Models for longer cation chains ([HMIm]^+^) often included descriptors related to more complex interaction patterns compared to shorter chains ([EMIm]^+^, [BMIm]^+^), which is likely due to additional interactions present with the longer alkyl side chain. Generally, in the modeled data sets, the log *K* values for the same IL increase in the following order: cyclohexane, benzene, methanol (Figure 3). Methanol has stronger molecular interactions toward its IL counterparts, which can be explained by hydrogen bonding and charge separation allowing for dipolar interactions.

### Applicability Domain and Outliers

**Figure 5:**
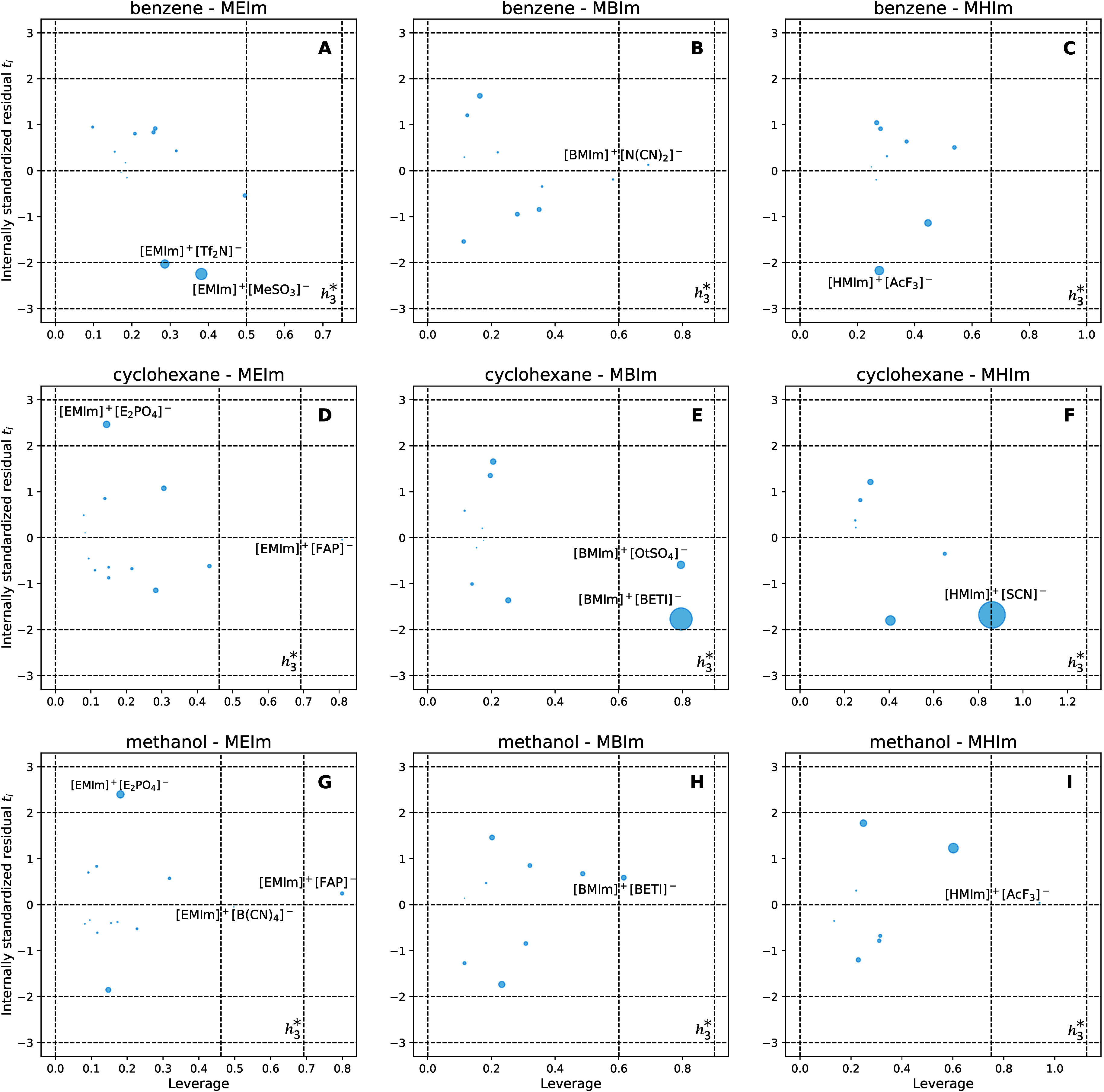
Influence plots with Cook’s distance estimate. The size of the data points on this plot is proportional to Cook’s distance. Horizontal and vertical lines show the thresholds for identifying moderate and high influence compounds.

The applicability domain of QSPR models was evaluated using an influence plot (Figure 5), which showed standardized residuals against leverage values. Most Cook’s distance values were below 1.0, indicating a need for further analysis of those exceeding this threshold. Box-swarm plots with experimental log *K* values (Figure 4) characterize the distribution of training set data and the predicted log *K* values (Table 1) highlighted areas in molecular space that could benefit from additional data points.

For the benzene-[EMIm]^+^ model, the ionic liquid [EMIm]^+^[MeSO_3_]^−^ was identified as a moderate outlier, exceeding the 2 sigma residual threshold (Figure 5A). Despite its outlying experimental log *K* value of 2.297 (Table 1) and the presence of similar functional groups in other compounds, [MeSO_3_]^−^ is the smallest SO_3_^–^-containing anion. Conversely, ionic liquid [EMIm]^+^[Tf_2_N]^−^ showed a moderate residual and is the largest, most complex anion. Log *K* values (Figure 3A) and descriptor values (Figure 4A) were evenly distributed. Considering the distribution of training set data (Figure 4), incorporating experimental log *K* values for benzene with [EMIm]^+^[BETI]^−^, [EMIm]^+^[OtSO_4_]^−^, and [EMIm]^+^[FAP]^−^ could enhance the model’s range and to further verify its applicability (see Table 1 for predicted values and Figure S20 for the expected location of these data points).

The benzene-[BMIm]^+^ model included [BMIm]^+^[N(CN)_2_]^−^ as a moderate leverage but low influence point (Figure 5B). Despite some ILs having outlying descriptor values (Figure 4B), no influential outliers were identified by the Cook’s distance. The limited range of experimental log *K* values (Table 1) suggests expanding the molecular space could improve model accuracy. The inclusion of [BMIm]^+^[MeSO_3_]^−^ and [BMIm]^+^[B(CN)_4_]^−^ could enhance the range of the data set substantially (Table 1 (predicted values marked with asterisk) and Figure S20 (predicted values in orange).

In the benzene-[HMIm]^+^ model, [HMIm]^+^[AcF_3_]^−^ was a moderate outlier, slightly exceeding the 2 sigma threshold (Figure 5C). It is a unique IL with the COO^–^ group and has the lowest log *K* value for benzene (Table 1). Descriptor plots (Figure 4C) showed clustering and gaps, likely influencing the model. Obtaining experimental log *K* values for benzene with [HMIm]^+^[MeSO_3_]^−^ and [HMIm]^+^[TDI]^−^ could fill gaps, while [HMIm]^+^[(MeO)(H)PO_2_]^−^ and [HMIm]^+^[EtSO_4_]^−^ might expand the model’s range (Figure S20, Table 1).

The cyclohexane-[EMIm]^+^ model had [EMIm]^+^[E_2_PO_4_]^−^ as a moderate residual outlier (Figure 5D) and [EMIm]^+^[FAP]^−^ as a moderate leverage point. Despite well-distributed descriptor values, two outliers for AETA_eta_R were noted (Figure 4D). The log *K* value with [EMIm]^+^[E_2_PO_4_]^−^ was outlying, and predictions suggest that including experimental log *K* values for benzene with [HMIm]^+^[PF_6_]^−^, [HMIm]^+^[TDI]^−^, [HMIm]^+^[BETI]^−^, and [HMIm]^+^[OtSO_4_]^−^ could improve the model (Figure S20, Table 1).

The cyclohexane-[BMIm]^+^ model had [BMIm]^+^[BETI]^−^ as an influential outlier and [BMIm]^+^[OtSO_4_]^−^ as a moderate leverage point (Figure 5E). The analysis of ATS3m descriptor distribution indicated a large clustering of its values and the presence of outliers (Figure 4E). The model could benefit from experimental log *K* values for cyclohexane with [BMIm]^+^[B(CN)_4_]^−^ and [BMIm]^+^[FAP]^−^ (Figure S20, Table 1).

In the cyclohexane-[HMIm]^+^ model, [HMIm]^+^[SCN]^−^ was identified as an influential data point (Figure 5F), with an extreme log *K* value for cyclohexane (Table 1) and with the smallest anion size. Predicted log *K* values for cyclohexane with [AcF_3_]^−^, [BETI]^−^, [MeSO_3_]^−^, and [N(CN)_2_]^−^ could expand the experimental value distribution for further modeling (Figure S20, Table 1).

The methanol-[EMIm]^+^ model’s outlier, [EMIm]^+^[E_2_PO_4_]^−^ (Figure 5G), also appeared in the cyclohexane-[EMIm]^+^ model. Its extreme log *K* value for methanol (Table 1) and the presence of clustering in descriptor distributions (Figure 4G) suggested difficulty in extrapolation. Extending experimental log *K* values with [BMIm]^+^[FAP]^−^ could improve the model, especially considering its lower values in other models (Figure S20, Table 1).

For the methanol-[BMIm]^+^ model, [BMIm]^+^[BETI]^−^ was a moderate leverage point (Figure 5H), which is also an outlier in nHetero descriptor values (Figure 4H). The model’s range and accuracy might be enhanced by including experimental log *K* values for methanol with [BMIm]^+^[FAP]^−^ (Figure S20, Table 1).

Lastly, the methanol-[HMIm]^+^ model had [HMIm]^+^[AcF_3_]^−^ as a moderate leverage point (Figure 5I), unique for its COO– group and high log *K* value (Table 1). Additional measurements with larger anions like [BETI]^−^ could expand the model (Figure S20, Table 1).

Overall, outliers in the experimental vs predicted values plots (Figure 3) highlighted the limitations of experimental log *K* data on the applicability domain. However, the analysis showed that even small enhancements to the availability of experimental data, particularly for larger anions, could improve the models (see examples in Figure S20). Analysis on residuals showed that less than half the models contained a single data point with a residual amplitude above 2.0, i.e. moderate outlier, aligning with expectations of the normal distribution of the given data set sizes (95.5% of sample >2.0 and 99.8% > 3.0).

## Conclusion

This study rigorously tested the hypothesis that the structure of the anionic part of ILs significantly influences the gas-IL partition coefficient (log *K*), and that machine learning approaches can effectively model this relationship. The findings confirm the hypothesis, demonstrating a clear correlation between the anion structure and log *K* values measured for the solutes. Utilizing multiple linear regression models augmented by machine learning techniques, the study successfully quantified the impact of varying anionic structures on the partitioning behavior of benzene, cyclohexane, and methanol in ILs with fixed cationic components.

The developed models and the molecular descriptors they contain highlight the different effects of anionic structural components on log *K* values. For methanol, the systematically selected molecular descriptors indicated a stronger dependence on hydrogen bonding and Coulomb-dipolar solute -solvent interactions, in contrast with benzene and cyclohexane, where molecular descriptors were related to the dispersion forces that played a more significant role in molecular interactions on interfaces and bulk of gas-IL molecular system. This distinction underscores the specificity of IL-solute interactions and the necessity to consider the unique characteristics of both solute and IL components in designing application-tailored ILs. The fact that the molecular descriptors selected for the models with the help of machine learning solutions are in very good agreement with our general knowledge of possible mechanisms in the interaction between molecules, significantly increases the reliability of the derived models as a descriptive and predictive tool.

In addition, outlier identification and data analysis emphasize the need for diverse and comprehensive experimental data. The outliers identified in the models suggest potential areas for further empirical research, suggesting that expanding the data set, especially with larger anions, could increase the predictive accuracy of the models and extend their applicability.

In summary, the research confirms the initial hypothesis and provides concrete evidence of the significant role that anion structure of ILs plays in influencing the gas-IL partition of organic solutes on interfaces and in bulk in complex liquid media. These insights contribute valuable knowledge to the field of IL interfaces, especially in the context of their application as solvents and in other industrial processes. This study lays the foundation for future research to optimize IL properties for specific applications, guided by an improved understanding of the fundamental interactions. Considering the results of this and our two previous studies, it is extremely interesting to simultaneously consider the structure-related characteristics of all three components of the organic solute-IL molecular system in the modeling of log *K*.

## Supporting Information Available

Descriptor values and log *K* values for anions in the benzene-[EMIm]^+^ data set (Figures S1 and S2), in the benzene-[BMIm]^+^ data set (Figures S3 and S4), benzene-[HMIm]^+^ data set (Figures S5 and S6), cyclohexane-[EMIm]^+^ data set (Figures S7 and S8), cyclohexane-[BMIm]^+^ data set (Figures S10 and S11), cyclohexane-[HMIm]^+^ data set (Figures S12 and S13), methanol-[EMIm]^+^ data set (Figures S14 and S15), methanol-[BMIm]^+^ data set (Figures S16 and S17) and methanol-[HMIm]^+^ data set (Figures S18 and S19); descriptor correlation matrix (Figure S9); experimental vs predicted log *K* plots for each model including anions with missing experimental data plotted as predicted vs predicted log *K* (Figure S20) (PDF)
