# Supplementary material for: Exploring the Influence of Ionic Liquid Anion Structure on Gas-Ionic Liquid Partition Coefficients of Organic Solutes Using Machine Learning
Source: Langmuir. 2024 Oct 30;40(45):23714–28. doi: 10.1021/acs.langmuir.4c02628 (PMC11562803; doi:10.1021/acs.langmuir.4c02628)
Supplement: Supplementary file 1 — la4c02628_si_001.pdf [file la4c02628_si_001.pdf]

Exploring the influence of ionic liquid anion structure on gas-ionic liquid partition coefficients of organic solutes using machine learning

Karl Marti Toots[1], Sulev Sild[1], Jaan Leis[1], William E. Acree[2], Uko Maran[1*]

[1] Department of Chemistry, University of Tartu, 14a Ravila Street, Tartu 50411, Estonia
[2] Department of Chemistry, University of North Texas, 1155 Union Circle Drive #305070, Denton, TX, 76203-5017, USA

* Corresponding author

Supplementary Information

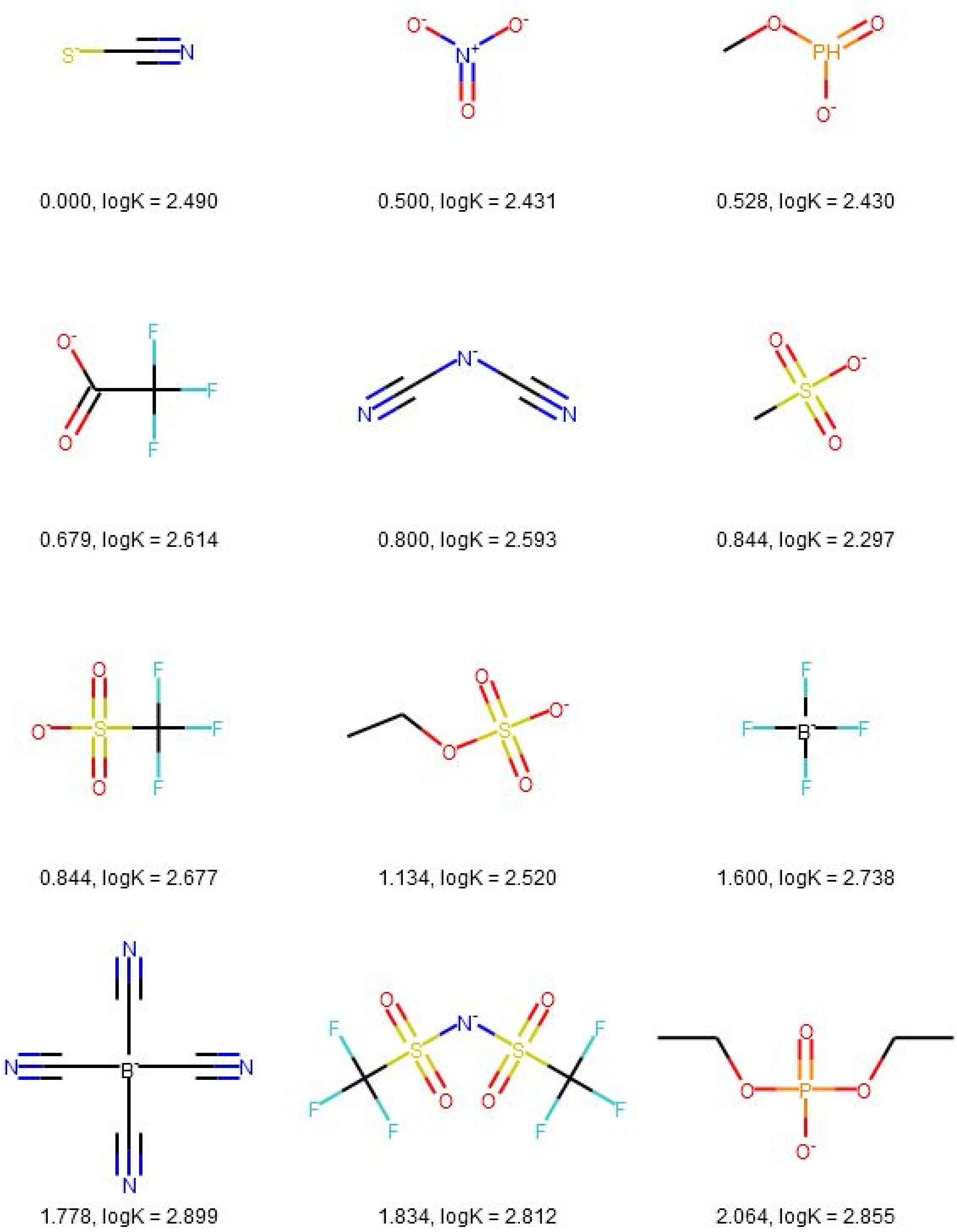

Figure S1. CIC1 descriptor values and log K with anions in the benzene-$[EMIm]^+$ dataset.

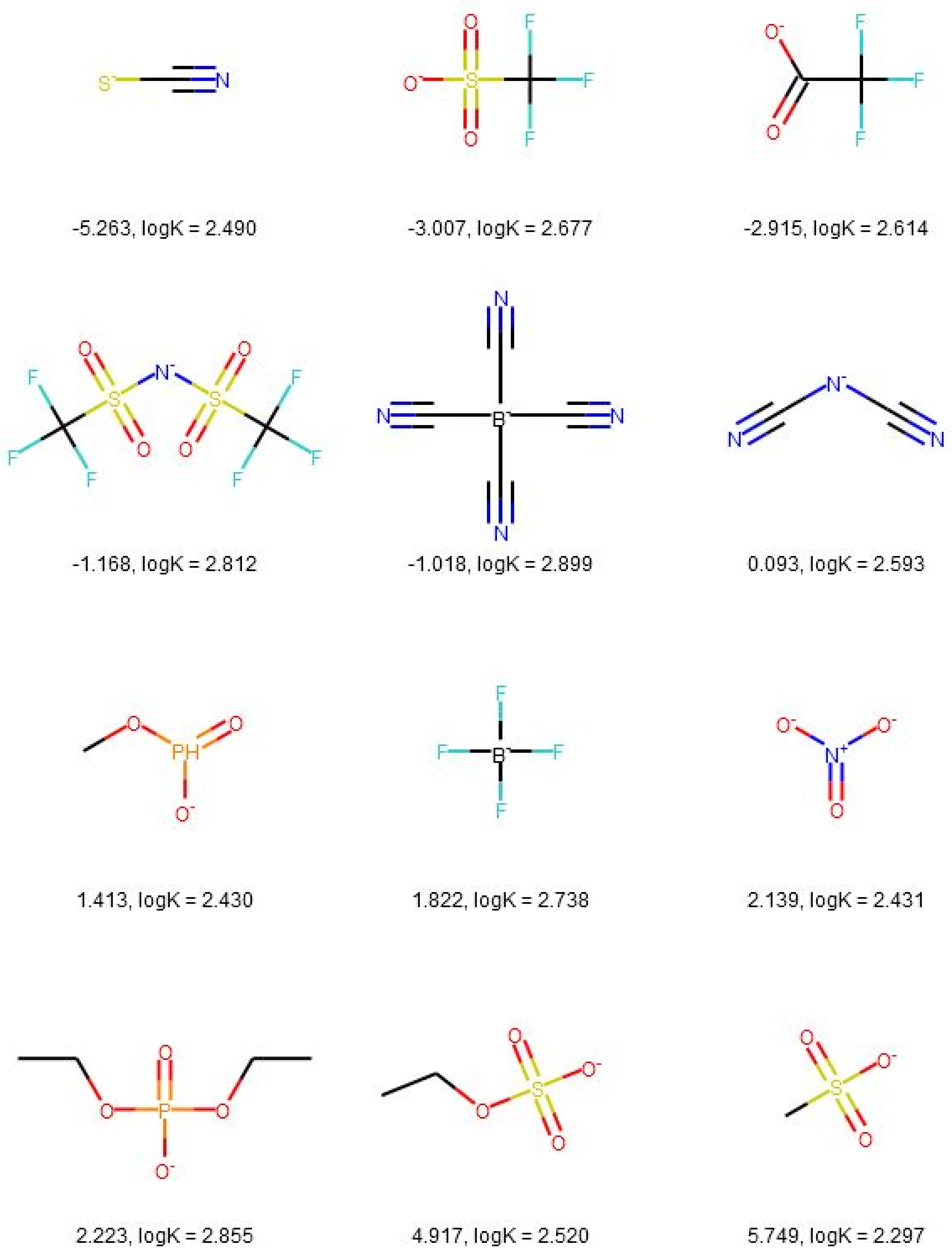

Figure S2. AATSC2s descriptor values and log K with anions in the benzene-$[EMIm]^+$ dataset.

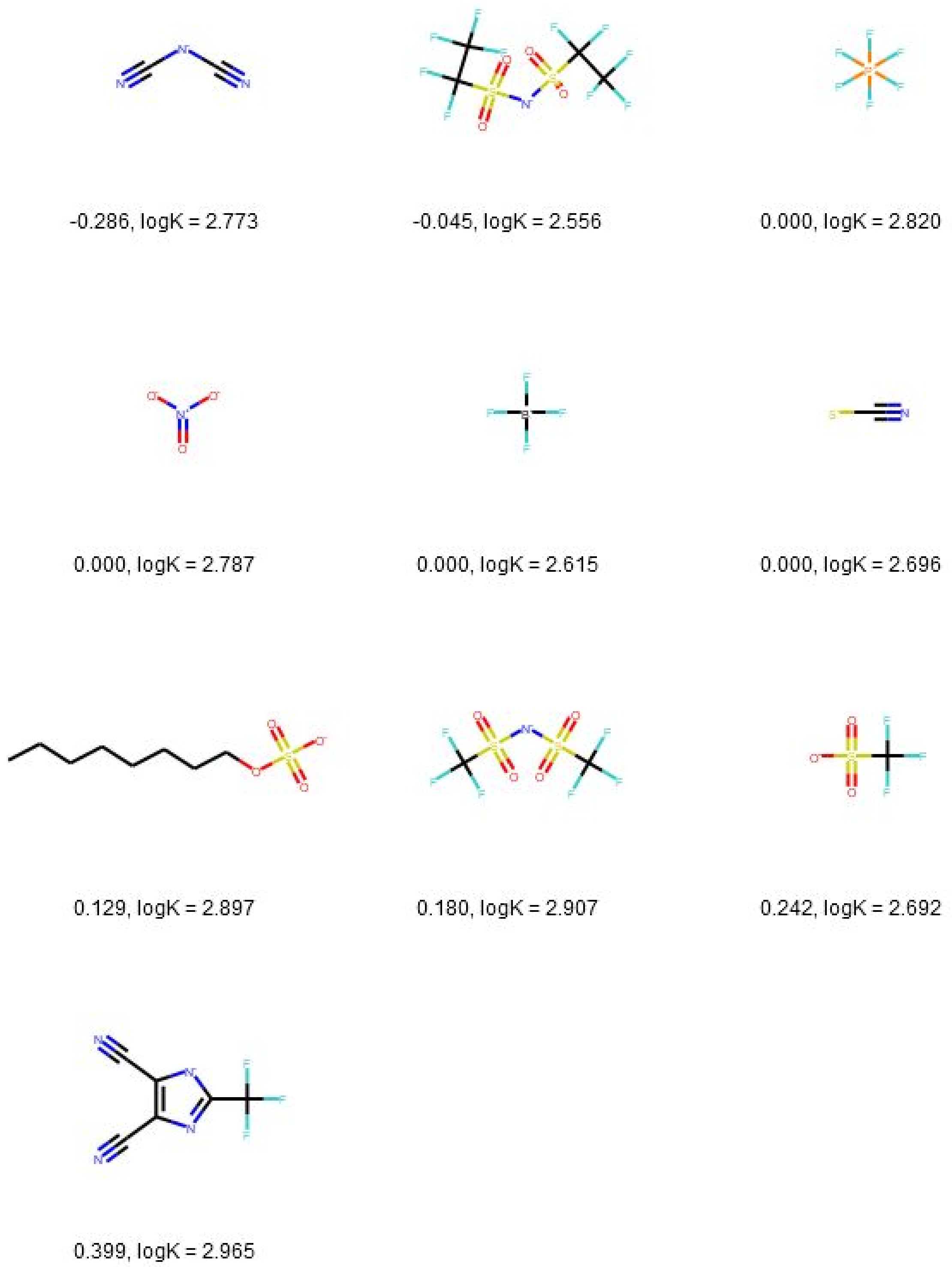

Figure S3. MATS3dv descriptor values and log K with anions in the benzene-$[BMIm]^{+}$ dataset.

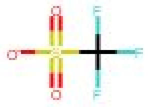

-0.174, logK = 2.692

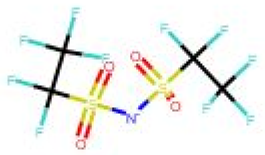

-0.165, logK = 2.556

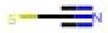

-0.059, logK = 2.696

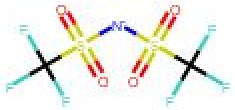

0.013, logK = 2.907

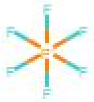

0.167, logK = 2.820

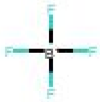

0.250, logK = 2.615

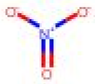

0.333, logK = 2.787

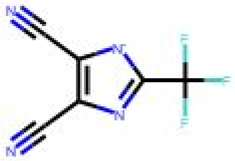

0.359, logK = 2.965

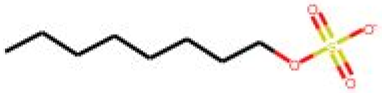

0.701, logK = 2.897

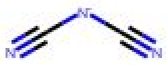

0.944, logK = 2.773

Figure S4. MATS2se descriptor values and log K with anions in the benzene-$[BMIm]^+$ dataset.

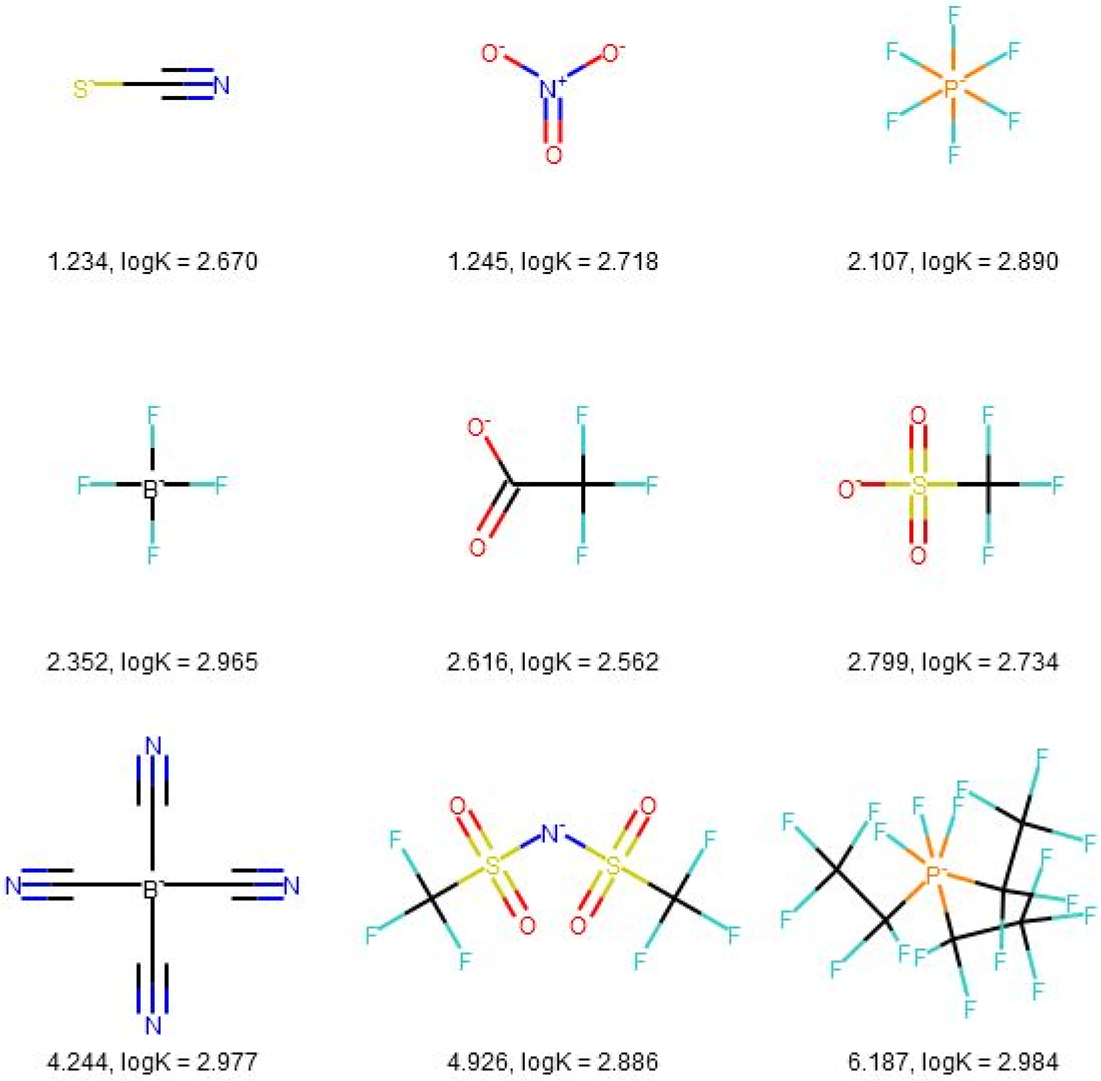

Figure S5. SpMAD_Dzi descriptor values and log K with anions in the benzene-$[HMIm]^+$ dataset.

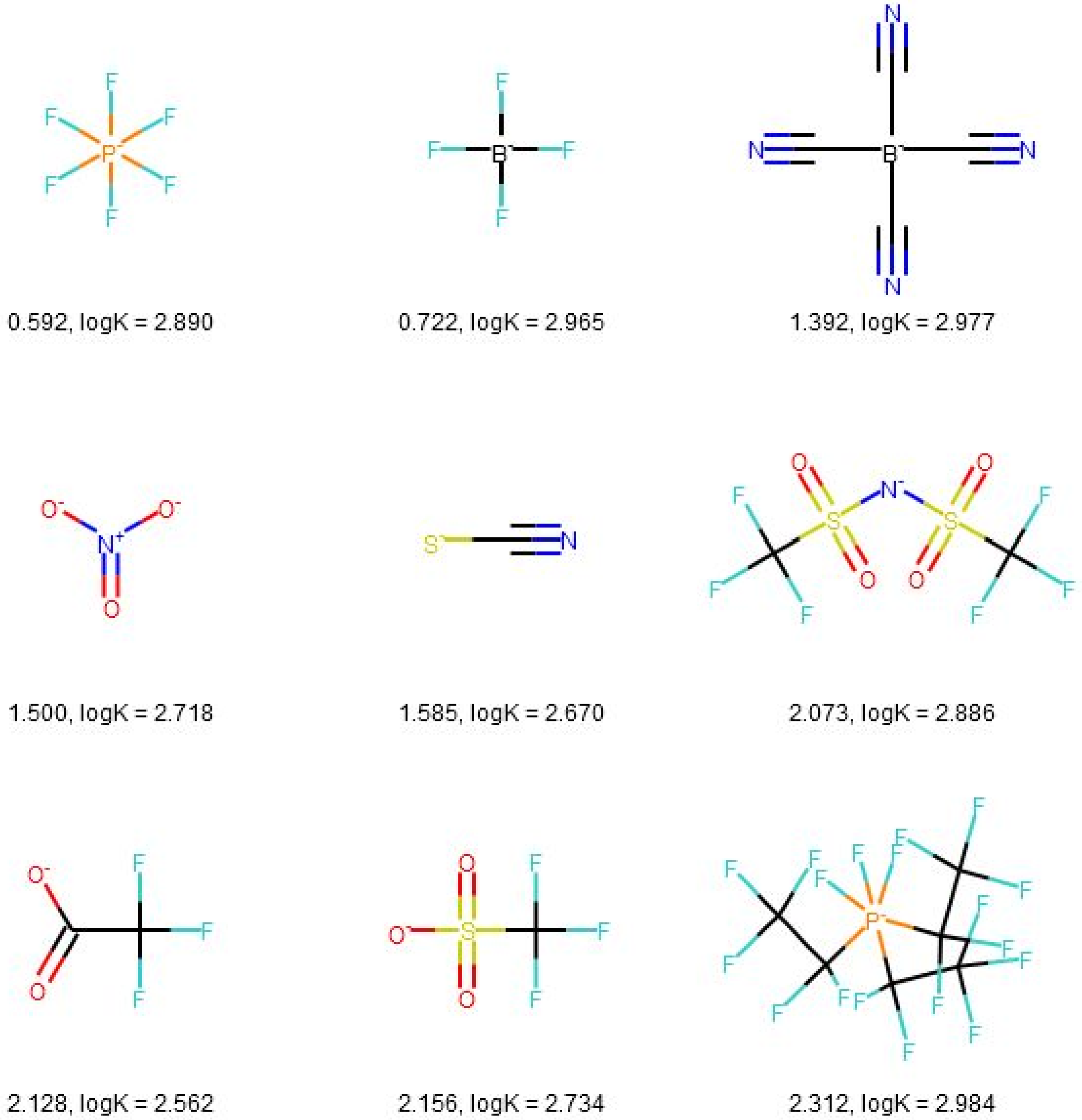

Figure S6. IC2 descriptor values and log K with anions in the benzene-$[HMIm]^+$ dataset.

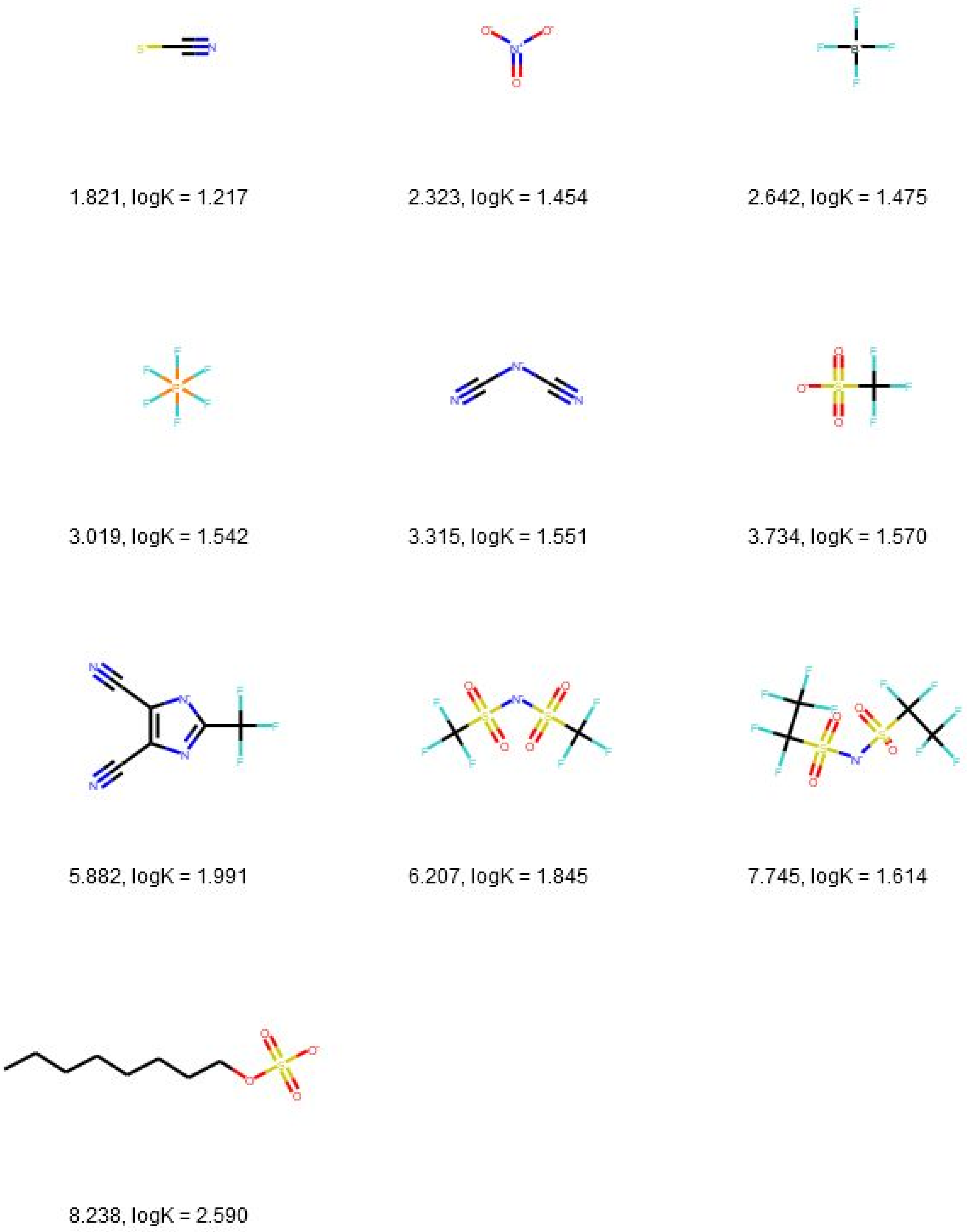

Figure S7. SpMAD_Dzm descriptor values and log *K* with anions in the cyclohexane-$[EMIm]^+$ dataset.

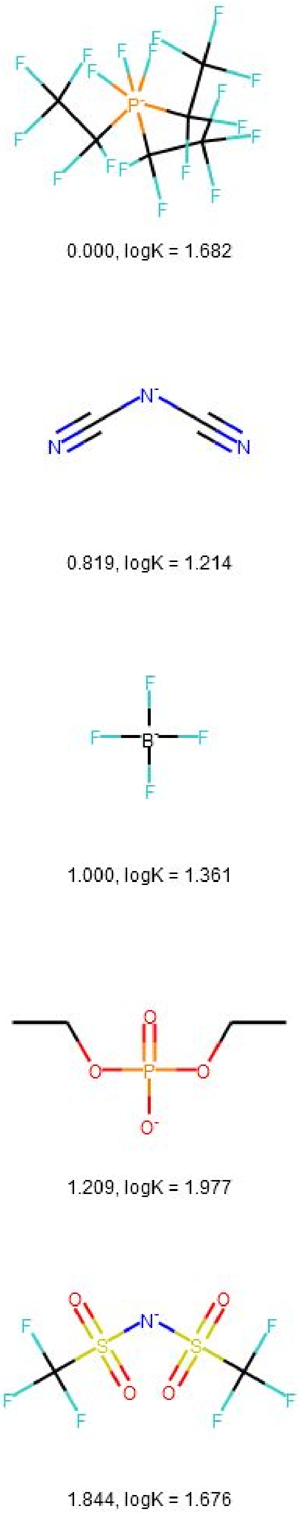

Figure S8. AETA_eta_R descriptor values and log *K* with anions in the cyclohexane-$[EMIm]^+$ dataset.

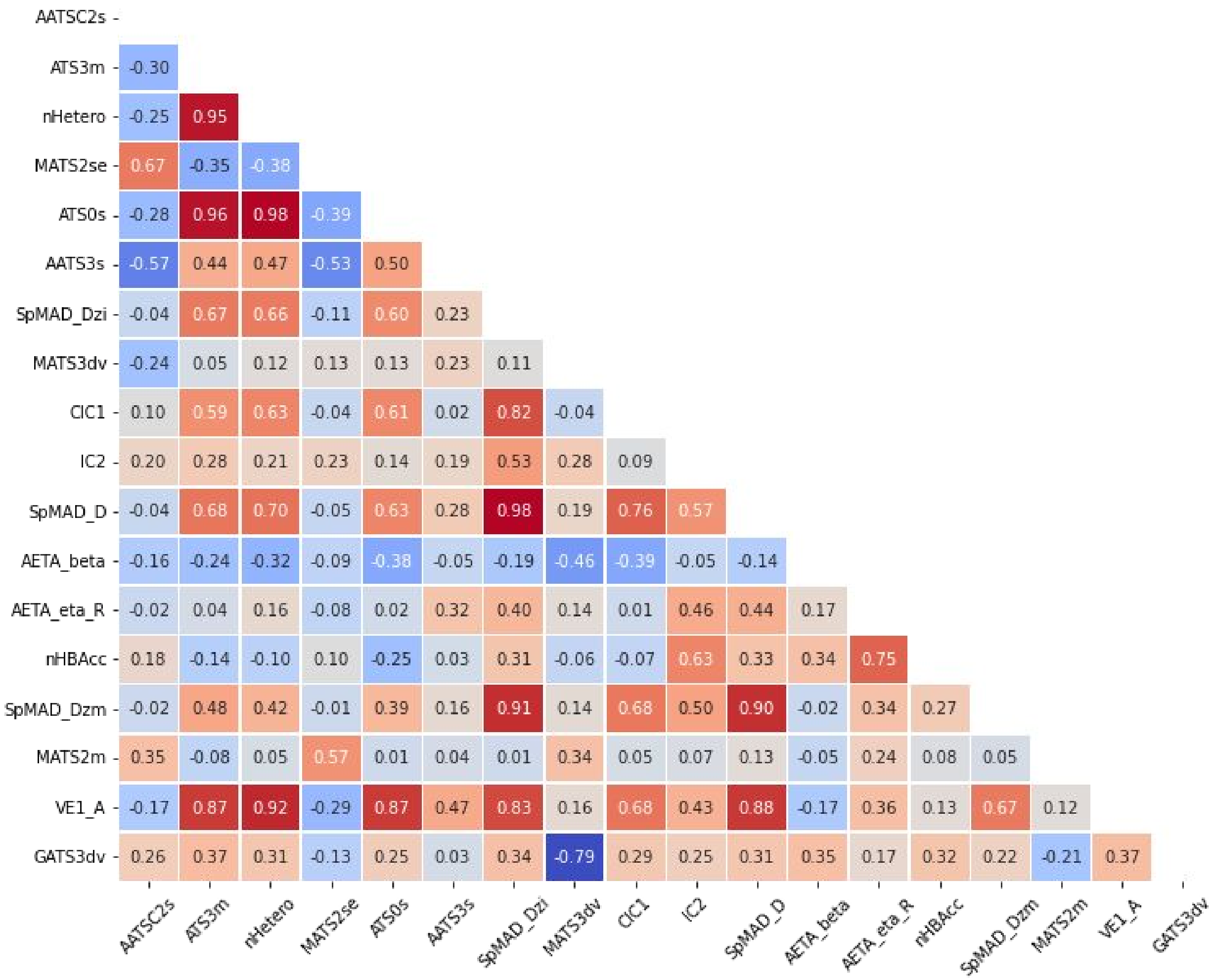

Figure S9. Descriptor correlation matrix

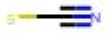

1.821, logK = 1.217

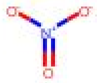

2.323, logK = 1.454

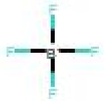

2.642, logK = 1.475

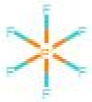

3.019, logK = 1.542

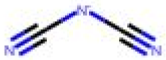

3.315, logK = 1.551

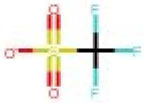

3.734, logK = 1.570

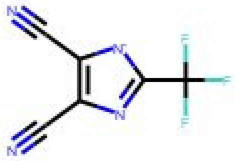

5.882, logK = 1.991

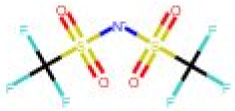

6.207, logK = 1.845

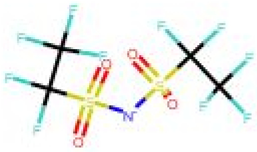

7.745, logK = 1.614

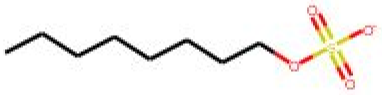

8.238, logK = 2.590

Figure S10. SpMAD_D descriptor values and log K with anions in the cyclohexane-[BMIm]$^+$ dataset.

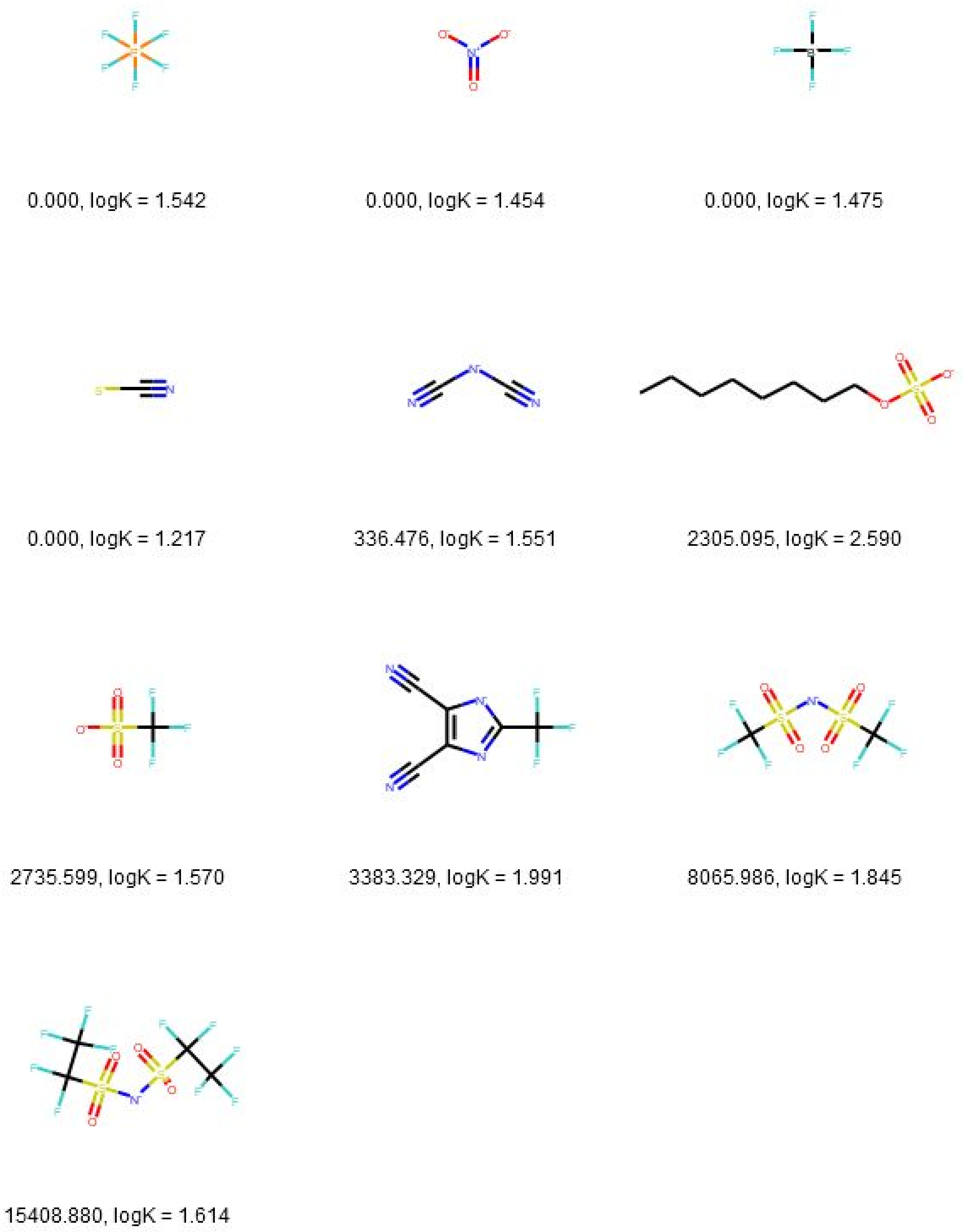

Figure S11. ATS3m descriptor values and log K with anions in the cyclohexane-$[BMIm]^+$ dataset.

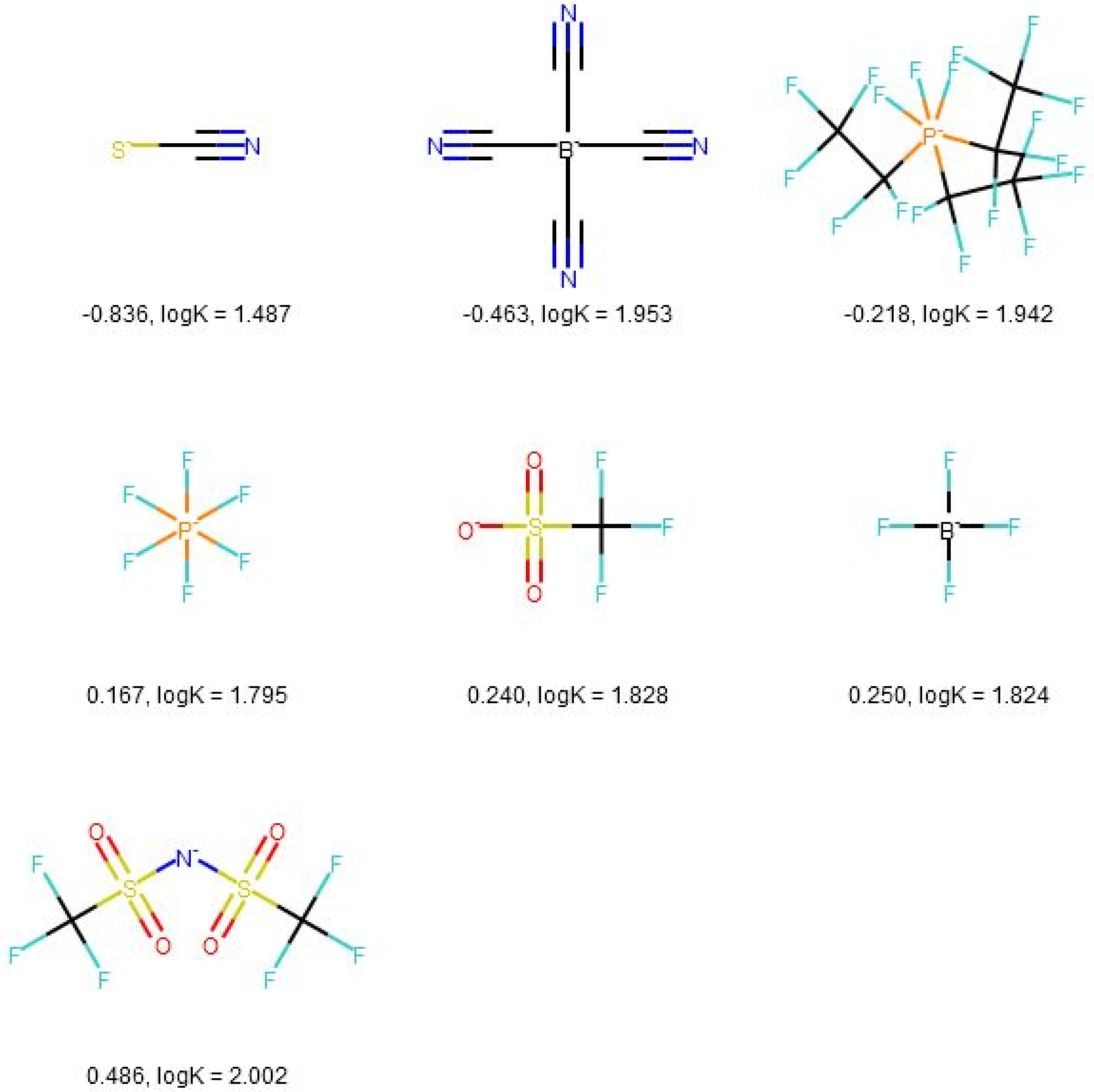

Figure S12. MATS2m descriptor values and log K with anions in the cyclohexane-$[HMIm]^+$ dataset.

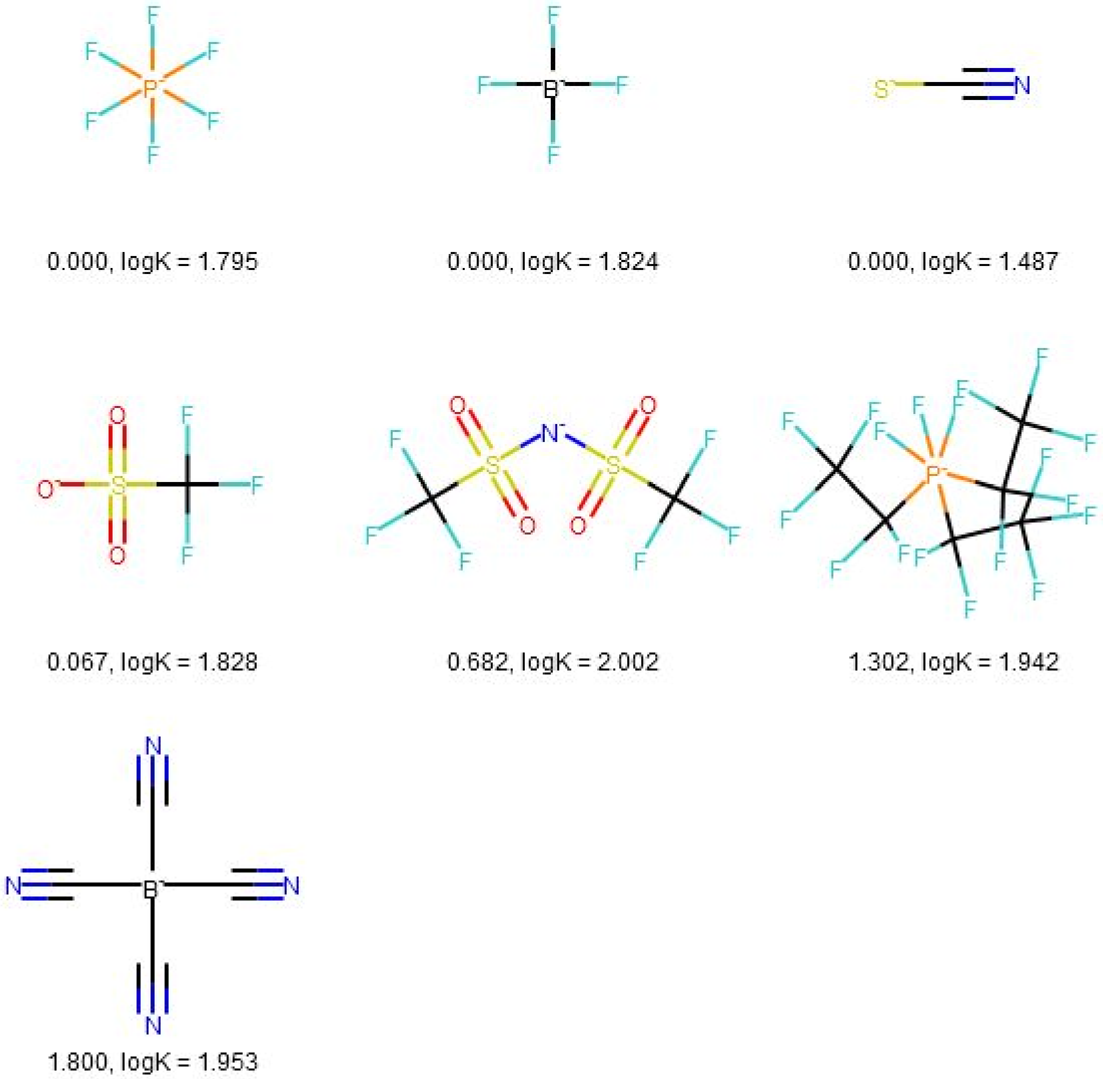

Figure S13. GATS3dv descriptor values and log K with anions in the cyclohexane-[HMIm]$^{+}$ dataset.

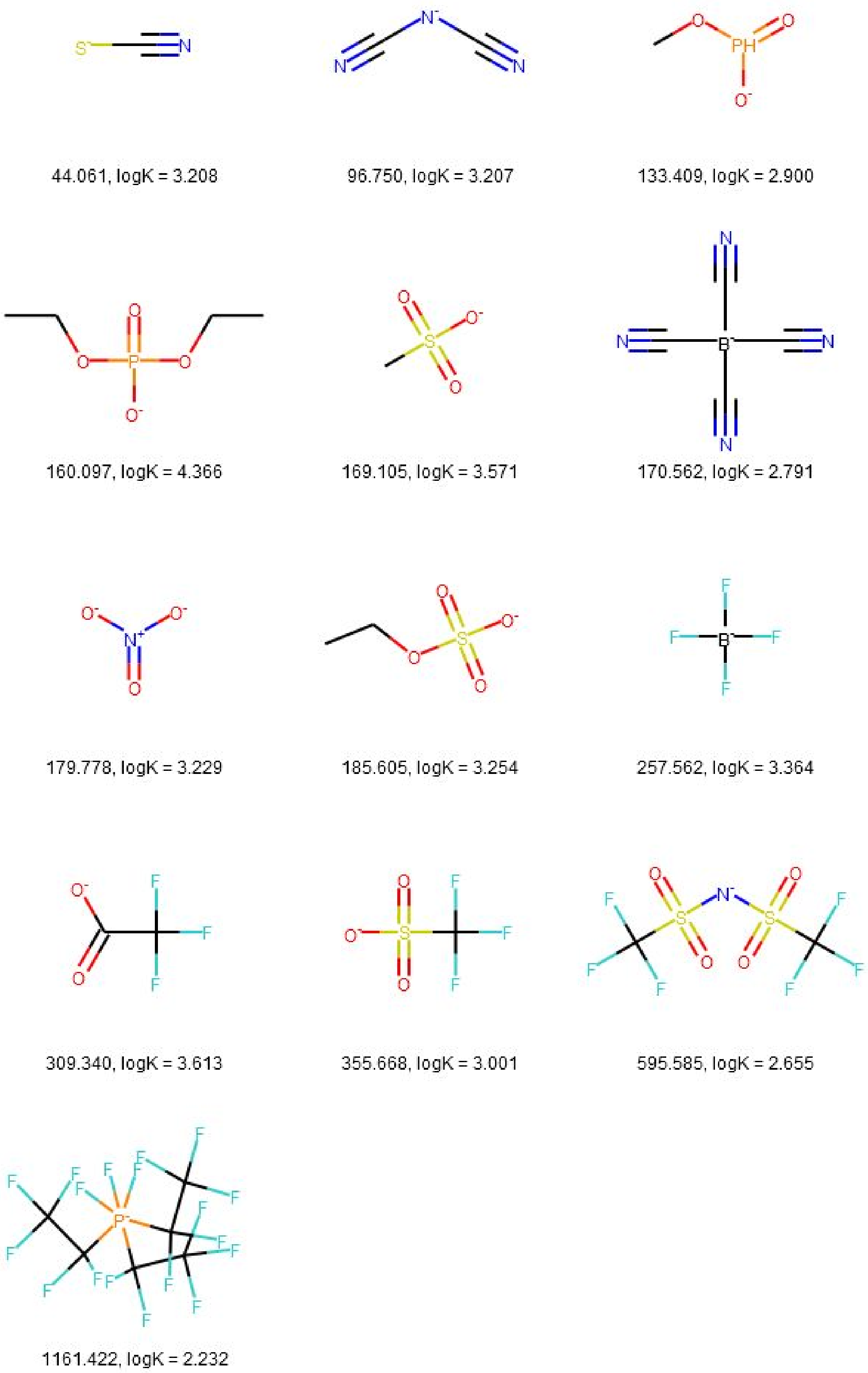

Figure S14. ATS0s descriptor values and log K with anions in the methanol-$[EMIm]^+$ dataset.

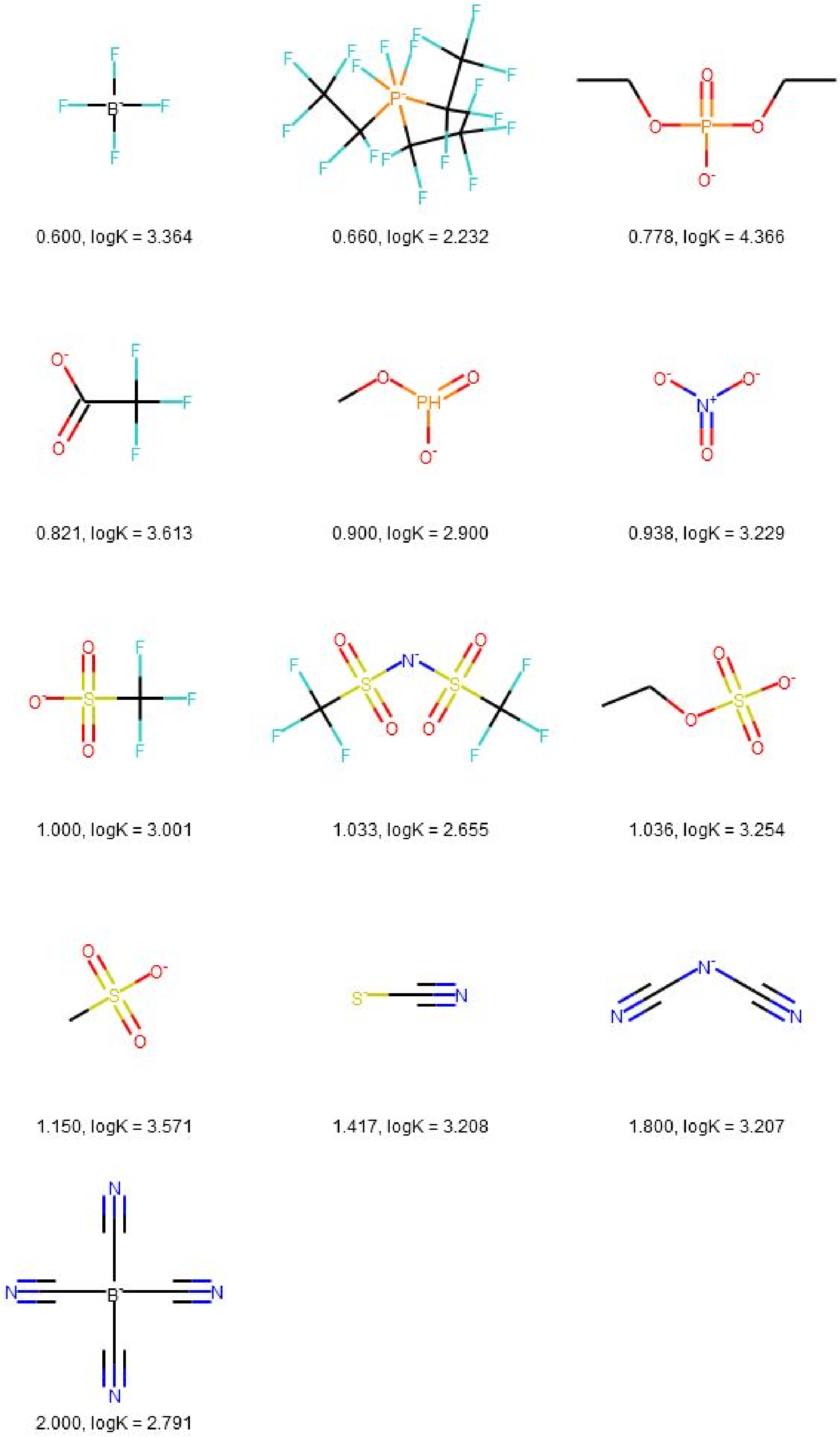

Figure S15. AETA_beta descriptor values and log K with anions in the methanol-[EMIm]$^{+}$ dataset.

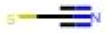

2.000, logK = 3.280

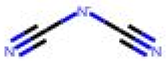

3.000, logK = 3.297

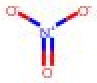

4.000, logK = 3.416

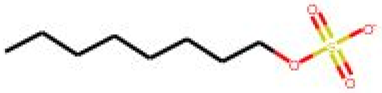

5.000, logK = 3.448

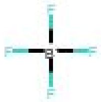

5.000, logK = 2.749

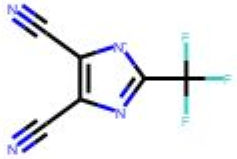

7.000, logK = 3.056

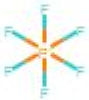

7.000, logK = 2.583

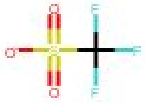

7.000, logK = 2.978

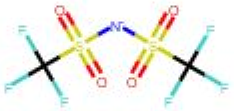

13.000, logK = 2.589

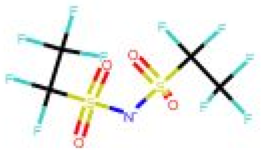

17.000, logK = 2.263

Figure S16. nHetero descriptor values and log K with anions in the methanol-[BMIm]$^+$ dataset.

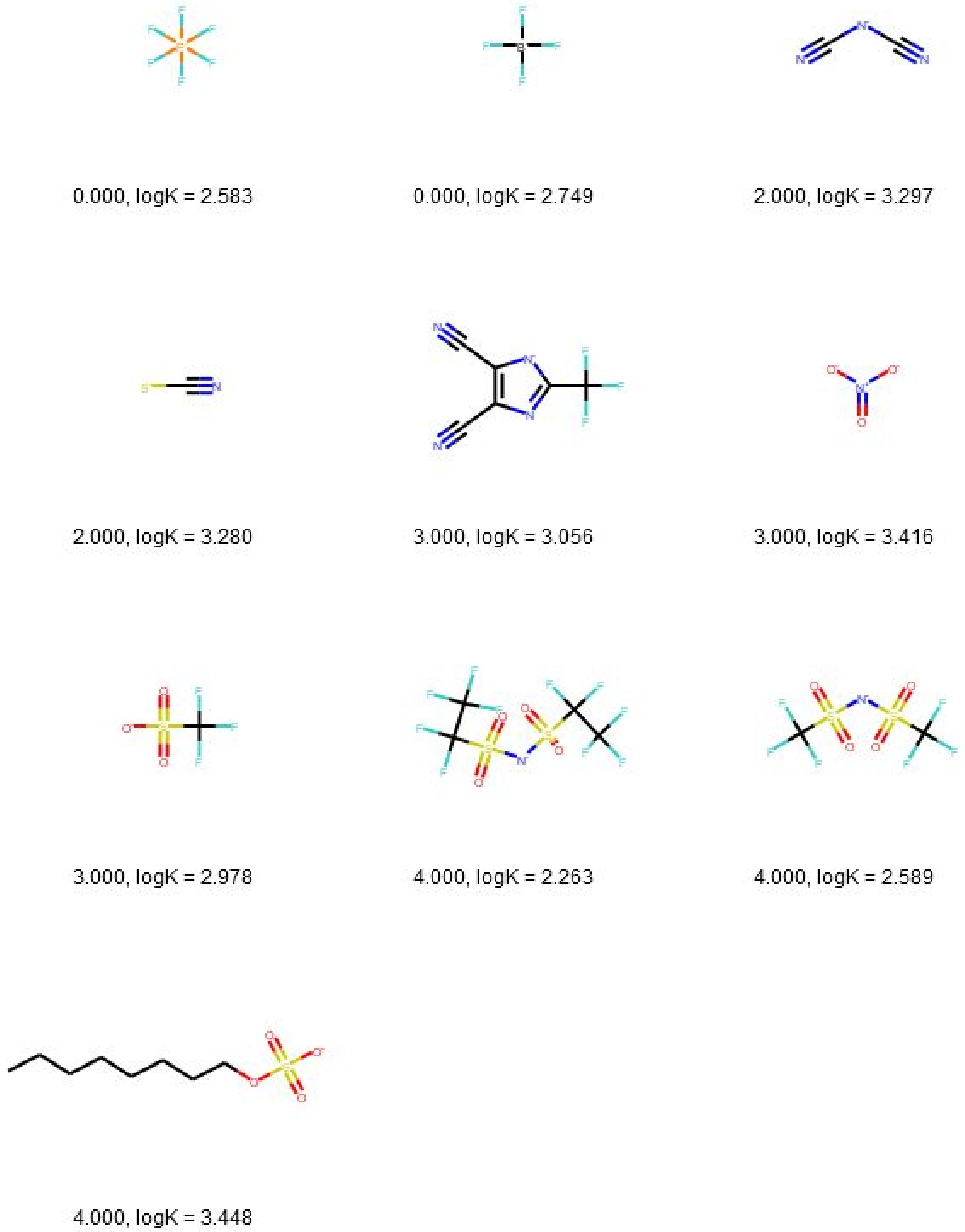

Figure S17. nHBAcc descriptor values and log K with anions in the methanol-$[BMIm]^+$ dataset.

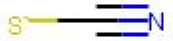

1.707, logK = 3.101

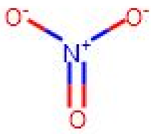

1.932, logK = 3.273

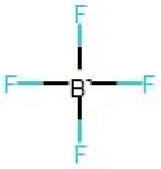

2.121, logK = 2.937

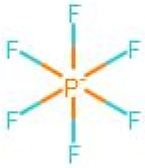

2.439, logK = 2.500

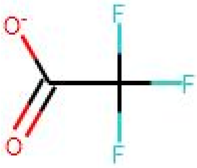

2.454, logK = 3.795

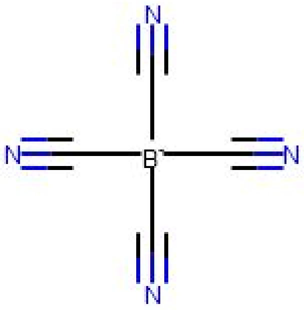

2.679, logK = 2.728

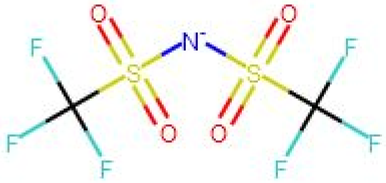

3.500, logK = 2.478

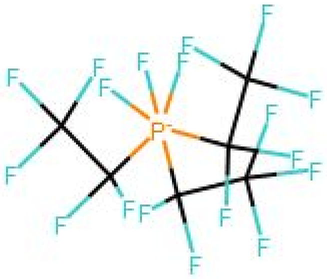

4.016, logK = 2.333

Figure S18. VE1_A descriptor values and log *K* with anions in the methanol-$[HMIm]^+$ dataset.

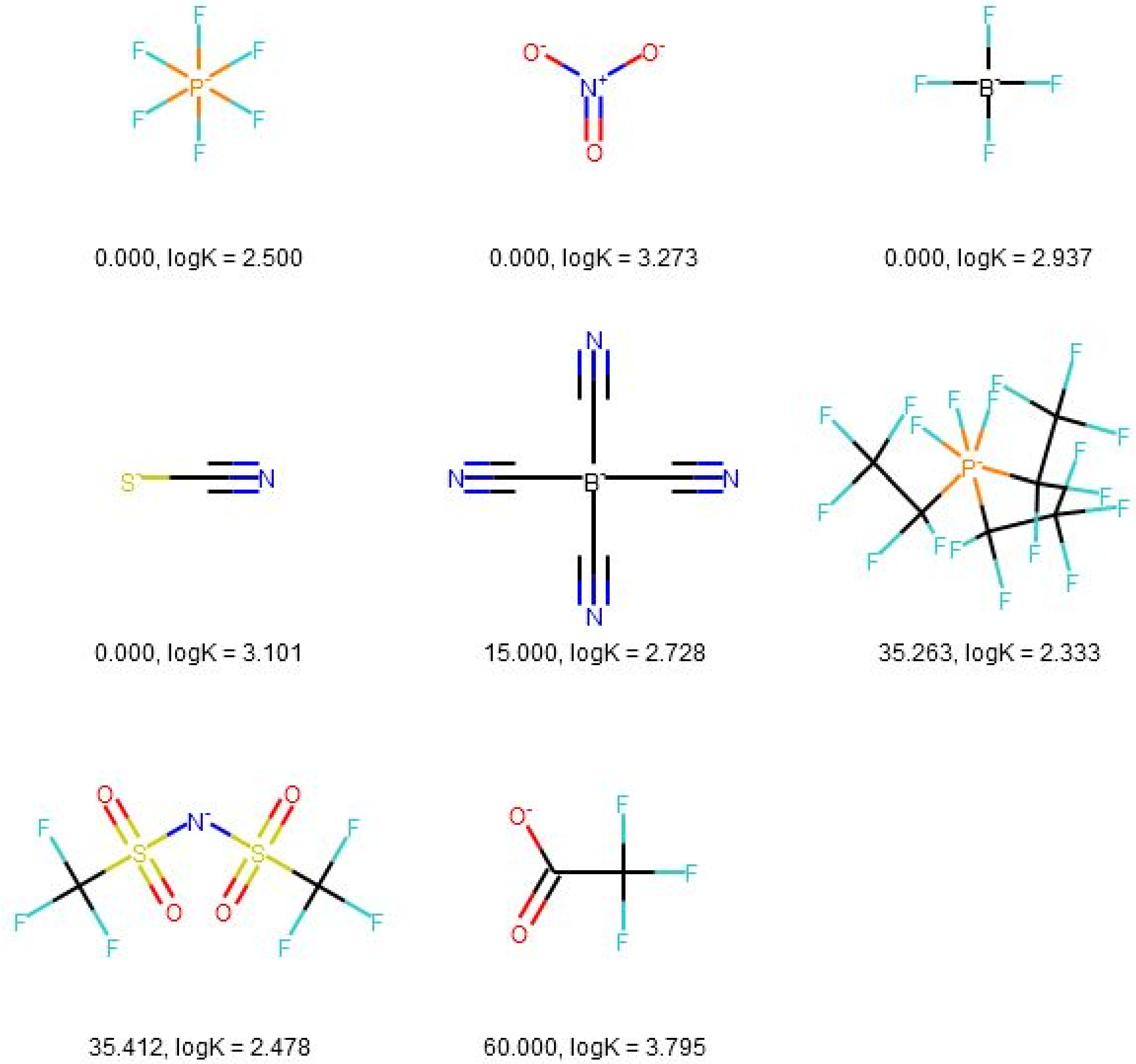

Figure S19. AATS3s descriptor values and log K with anions in the methanol-$[HMIm]^+$ dataset.

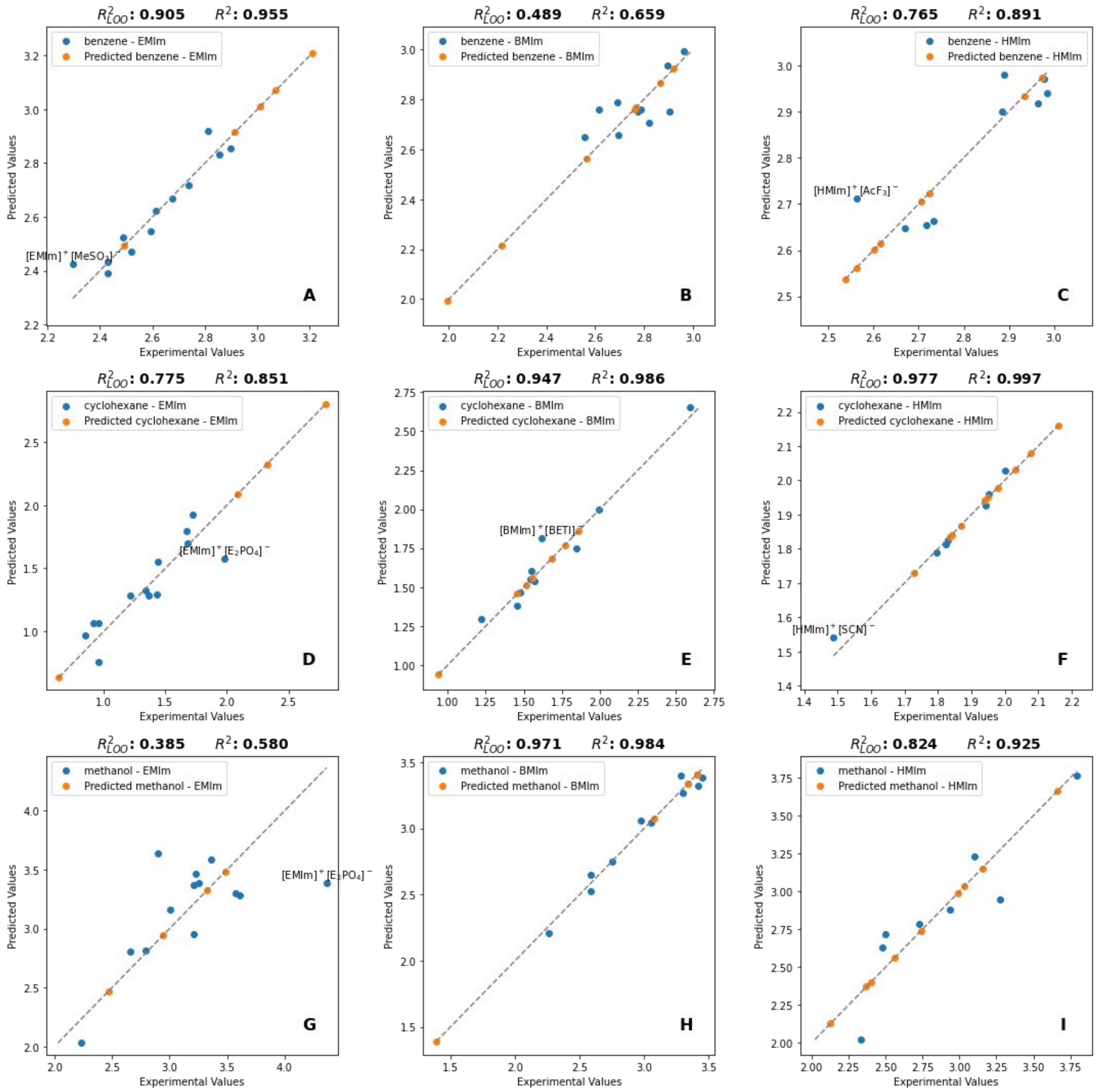

Figure S20. Experimental *vs* predicted log *K* plots for each model (blue) including anions with missing experimental data plotted as predicted *vs* predicted log *K* (orange).
